# Externally triggered drug delivery systems

**DOI:** 10.1016/j.smaim.2024.08.004

**Published:** 2024-08-30

**Authors:** Huiyang Hu, Prabhakar Busa, Yue Zhao, Chao Zhao

**Affiliations:** a Department of Chemical and Biological Engineering, University of Alabama, Tuscaloosa, AL, 35487, USA; b School of Computer Science, College of Science, Mathematics and Technology, Wenzhou-Kean University, Wenzhou, China; c Center for Convergent Biosciences and Medicine, University of Alabama, Tuscaloosa, AL, 35487, USA

**Keywords:** External stimuli-responsive, On-demand drug delivery, Temperature-sensitive polymers, Nanoparticles, Hydrogel

## Abstract

Externally triggered drug delivery systems empower patients or healthcare providers to utilize external stimuli to initiate drug release from implanted systems. This approach holds significant potential for clinical disease management, offering appealing features like enhanced patient adherence through the elimination of needles and medication reminders. Additionally, it facilitates personalized medicine by granting patients control over the timing, dosage, and duration of drug release. Moreover, it enables precise drug delivery to targeted locations where external stimuli are applied. Advances in materials science, nanotechnology, chemistry, and biology have been pivotal in driving the development of these systems. This review presents an overview of the progress in research on drug release systems responsive to external stimuli, such as light, ultrasound, magnetic fields, and temperature. It discusses the construction strategies of externally triggered drug delivery systems, the mechanisms governing triggered drug release, and their applications in disease management.

## 1. Introduction

Chronic disorders often require frequent treatment. However, needle injections can cause discomfort, leading to reduced patient compliance, while scheduling issues may result in missed doses of oral medications [1,2]. To address these challenges, sustained-release drug systems have been developed utilizing liposomes [3], nanoparticles [4], and gels as drug carriers [5]. Many of these systems passively release drug molecules to maintain therapeutic concentrations, extending drug release over weeks to months and reducing the need for frequent administration; thus, enhancing patient compliance [6]. However, these release systems typically exhibit a uniform pattern and lack precise control over drug release [7]. Drug release initiates upon administration, with the drug carrier primarily delaying release rather than regulating it based on the patient’s specific condition [8,9]. Additionally, there is usually a burst release in a drug sustained-release system, where a large quantity of drug is released initially, this high drug concentration may lead to biological toxicity. Following the burst release, the drug release rate quickly decreases, and the reduced drug concentrations may prevent effective treatment [7].

**Fig. 1.**
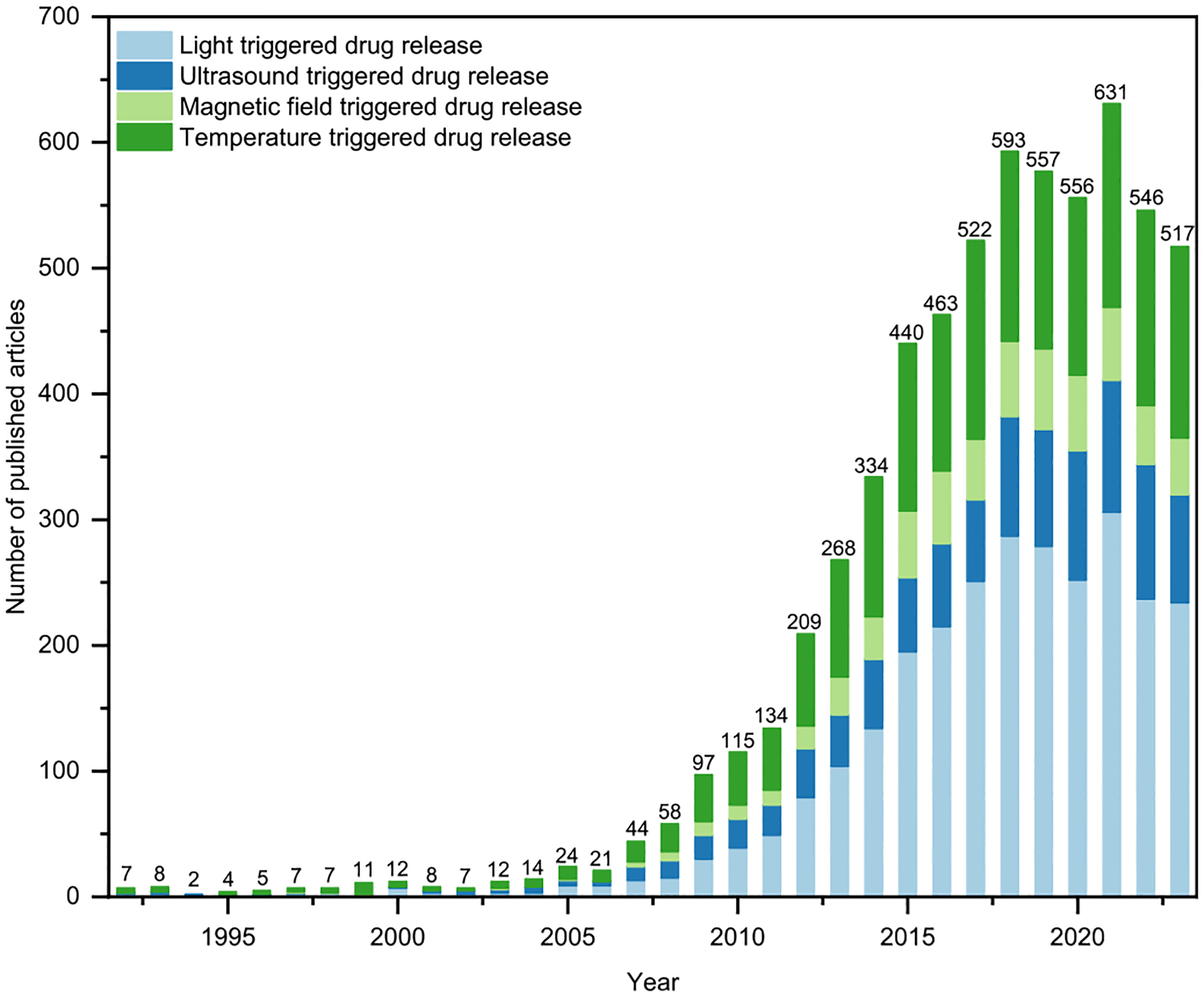
The average annual number of publications on externally triggered drug release systems. Data were obtained from Web of Science search for the term “light-triggered drug release”, “ultrasound-triggered drug release”, “magnetic field-triggered drug release”, and “temperature-triggered drug release systems”.

Intelligent drug delivery systems must demonstrate controlled drug release profiles and the capacity to offer diverse drug release rates at different time intervals. Externally triggered drug delivery systems can actively release drugs by altering the structure, morphology, and even the phase state of the drug carrier in response to external stimuli, such as light [10], ultrasound (US) [11], magnetic fields [12], temperature [13], etc. [14–18]. By regulating the dosage and duration of drug release in a non-invasive manner, patients can achieve on-demand drug release tailored to their pathology, thereby enhancing therapeutic effectiveness and reducing non-targeted injuries, crucial for specific local ailments like cancer, pain, diabetes, etc. [19]. Moreover, externally triggered drug delivery systems enable targeted drug release at the application site of stimuli, thereby reducing systemic drug distribution to enhance drug efficacy while minimizing side effects [20]. With these appealing attributes, the externally triggered drug delivery approach holds promise for managing clinical diseases. Considerable efforts have been devoted to developing intelligent stimulus-responsive drug release systems. As illustrated in Fig. 1, the number of articles published annually in this field has risen from an initial 7 to 517 between 1992 and 2023. Various stimulus-responsive vectors have been employed to address conditions such as pain [9,21], cancer [22], inflammatory arthritis [23], diabetes [24], etc.

## 2. Externally triggered drug delivery systems

### 2.1. Light-triggered drug release

Light, as an external stimulus, is attractive due to its temporal and spatial controllability, along with therapeutic convenience [10]. Light utilized to induce drug release is categorized into three wavelength ranges: ultraviolet (UV) (200–400 nm), visible (Vis) (400–700 nm), and near-infrared (NIR) (700–1000 nm) [25]. UV and Vis light possess higher energy levels, yet they encounter difficulty penetrating deep into tissues (less than 1 cm) due to absorption and scattering by oxygenated hemoglobin, hemoglobin, and melanin in tissues; thus, limiting their applicability [26]. Moreover, continuous exposure to UV, owing to its high energy, may lead to tissue damage and cancer [27]. NIR light with wavelengths exceeding 900 nm also faces poor penetration due to water absorption [28]. NIR light within the 650–900 nm range demonstrates enhanced tissue penetration, reaching depths of up to 10 cm [29], while causing less tissue damage, rendering it the most commonly employed photostimulation. In addition to triggering drug release, light also elicits therapeutic effects such as photothermal therapy (PTT) and photodynamic therapy (PDT), which assist drug molecules in combating diseases [30–33].

In recent years, numerous light-triggered drug release systems have been developed, as demonstrated in Table 1. Based on the different triggering principles, light-triggered drug release systems can be categorized into four categories: photodynamic effect (PDE)-triggered drug release systems, photothermal effect (PTE)-triggered drug release systems, photocleavage-triggered drug release systems, and upconversion nanoparticle (UCNP)-based light-triggered drug release systems.

#### 2.1.1. Mechanism of PDE-triggered drug release

The PDE can be defined as the effect of photosensitizers absorbing photons and being excited to the triplet state in PDT, generating reactive oxygen species (ROS) [67]. ROS can kill local tumor cells, thereby inhibiting tumor growth [68]. Additionally, for ROS-sensitive drug carriers, ROS may induce the breaking of chemical bonds, hydrophilicity conversion, increase membrane permeability, etc., thereby triggering the release of the encapsulated drug [69].

#### 2.1.2. PDE-triggered drug release systems

**Fig. 2.**
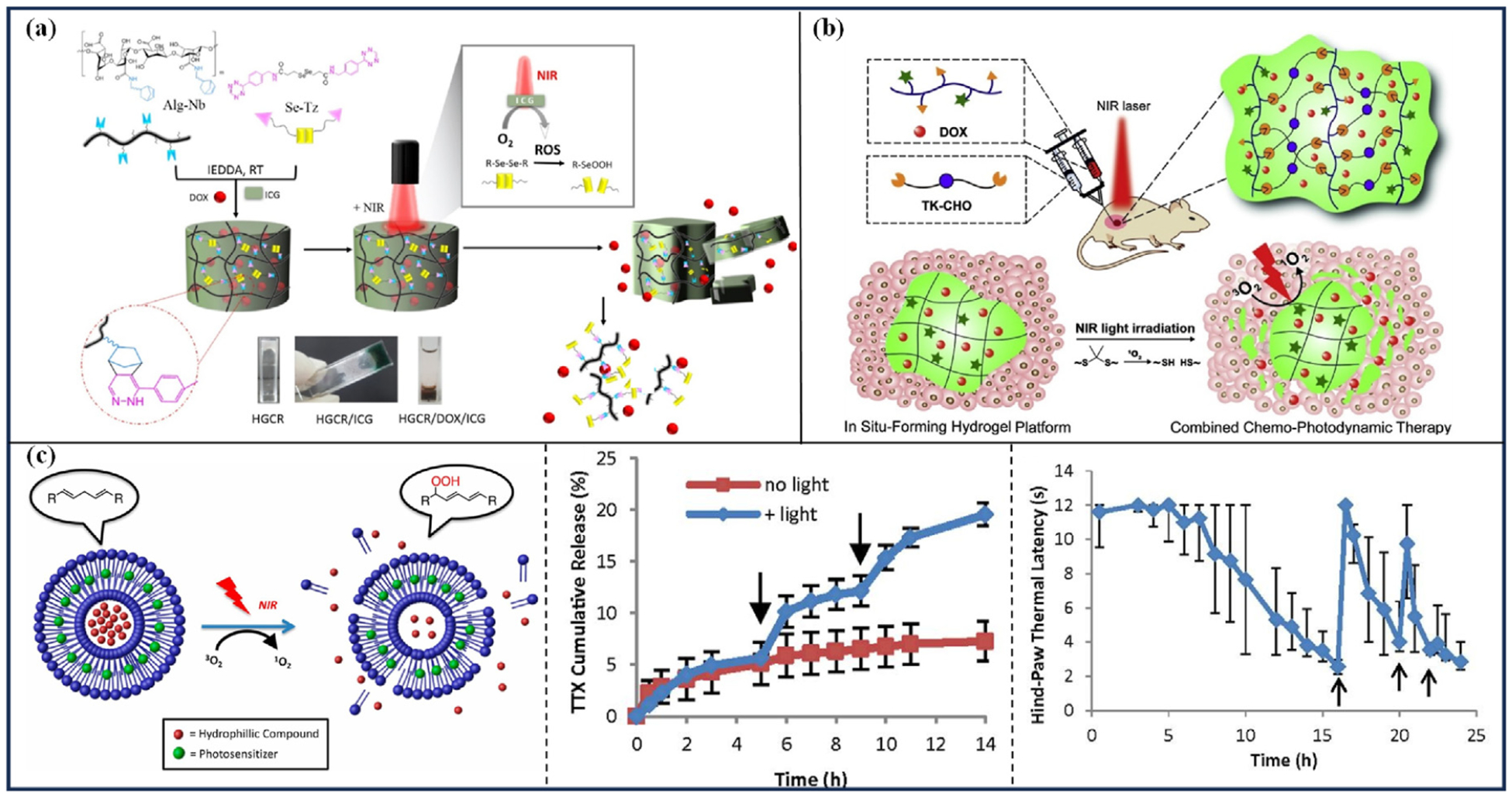
PDE-triggered drug release: (a) Schematic illustration of Alg hydrogels containing diselenide bonds for responsive drug release under NIR light irradiation. Reprinted with permission from Ref. [34]. Copyright (2019) Elsevier. (b) Schematic illustration of NIR light-triggered DOX release from ROS degradable hydrogels for tumor treatment. Reprinted with permission from Ref. [36]. Copyright (2020) Elsevier. (c) Left: Schematic illustration of photosensitizer-induced liposome peroxidation under NIR irradiation, which modifies the permeability of liposome membranes and facilitates the release of hydrophilic compounds for pain treatment. Middel: In vitro release profile of TTX subjected to NIR laser irradiation (730 nm, 50 mW/cm ^**2**^, 10 min) at the fifth and ninth hour. Right: Triggerability of the liposomes upon irradiation (730 nm, 330 mW/cm ^**2**^, 15 min) upon return of latency to baseline, at 16, 20, and 22 h. If the thermal latency exceeds 7 s, the anesthesia is deemed effective. Reprinted with permission from Ref. [42]. Copyright (2015) National Academy of Sciences.

The diselenide bond exhibits a dual redox reaction property and is readily oxidized to selenic acid by ROS, leading to the cleavage of the diselenide bond and the degradation of the drug carrier constructed with the diselenide bond [70]. Anugrah et al. [34] prepared NIR-responsive alginate (Alg) hydrogels loaded with the photosensitizer indocyanine green (ICG) and the anticancer drug doxorubicin (DOX), using cross-linking agents containing diselenide bonds (Fig. 2a). Under NIR light irradiation, ICG produces ROS, degrading the hydrogel matrix’s diselenide bonds, causing a gel-solution transition, and releasing DOX. Degradation investigations showed that NIR-exposed hydrogels underwent convenient de-crosslinking and fully dissolved within 16 days of NIR (808 nm, 2 W/cm ^2^, 15 min) exposure, with lower cross-linking densities degrading more swiftly. In vitro release experiments indicated that the hydrogels remained stable without stimulation, with only about 10 % of DOX released within 10 days. However, after five rounds of NIR irradiation (808 nm, 2 W/cm ^2^, 15 min), approximately 74 % of the DOX was released within 5 days, and 90 % was released within 10 days. At a power density of 1 W/cm ^2^, after five rounds of NIR irradiation, about 63 % of the DOX was released in 5 days, suggesting that NIR radiation can effectively release the drug in a controlled manner.

Zhu et al. [35] designed a ROS-sensitive hydrophilic TCPP@SeSe-CPT nanodrug by self-assembly of polyethylene glycol (PEG) -modified camptothecin (CPT) and endocyclic tetrakis (4-carboxyphenyl)porphyrin (TCPP, a hydrophobic photosensitizer). Under 660 nm laser irradiation, the ROS generated by TCPP disrupts the diselenide bond between CPT and the nanoparticles, leading to the rupture of the diselenide functional group in TCPP@SeSe-CPT and the release of CPT. In vitro experiments showed that after 5 and 10 min of NIR laser irradiation (660 nm, 300 mW/cm ^2^), the release of CPT was 35.6 ± 2.1 % and 82.4 ± 1.4 %, respectively. Moreover, the system exhibited significant cytotoxicity after light irradiation, which was much greater than the cytotoxicity of CPT alone. This result suggests that ROS-induced PDT and CPT synergistically enhanced cytotoxicity. Four hours after intravenous injection into HT29 hormonal nude mice, the fluorescence intensity peaked at the tumor site, while the other sites showed no fluorescence, demonstrating good targeting. The antitumor results indicated that NIR laser (660 nm, 300 mW/cm ^2^, 5 min) irradiation of TCPP@SeSe-CPT could effectively inhibit the tumor growth, and the tumor inhibition rate reached 74 % within 16 days.

Thioketal (TK) is easily cleaved by ROS, with its cleavage products being non-toxic [71]. Drug carriers utilizing TK as a building block or prodrugs employing TK as a linker can be stabilized in vivo while efficiently releasing guest drug molecules in the presence of ROS [72]. Xu et al. [36] developed an injectable NIR light-triggered degradation platform of hyaluronic acid (HA) hydrogel, wherein the photosensitizer protoporphyrin IX (PpIX) and DOX were loaded into the hydrogels (Fig. 2b). Under NIR irradiation, like ICG, PpIX generates a large amount of ROS, which triggers TK cleavage, resulting in hydrogel degradation and DOX release. About 61 % and 55 % of DOX in the hydrogel was released passively in 120 h, but after one or two irradiations with NIR light (633 nm, 50 mW/cm ^2^, 10 min), about 84 % and 94 % of DOX was released within 120 h. After loading gold nanorods (GNRs) as guest molecules into the hydrogel, only a small amount diffused from the hydrogel into the aqueous medium without laser irradiation, while light irradiation significantly increased GNR release. Anti-tumor tests were performed in 4T1 tumor-bearing mice. Compared with other controls, irradiation of NIR light (633 nm, 50 mW/cm ^2^, 10 min) every two days in the first nine days significantly inhibited tumor growth over the 18-day observation period, with the highest tumor ablation rate.

Zuo et al. [37] developed the tumor-specific nanotherapeutic drug IMTD by coupling ICG with the mannose-thioketal-DOX complex (MAN-TK-DOX). Under NIR laser irradiation, ICG molecules generate ROS to destroy the TK linker and accelerate the release of DOX, which induces cancer cell death. At the same time, PDT and PTT accompanying this process will also accelerate the death of cancer cells. After laser (808 nm, 0.5 W/cm ^2^, 10 min) irradiation, the nanoparticles released more than 80 % of the DOX after 70 h. However, without light irradiation, the DOX released within 72 h was less than 45 %. In the in vivo antitumor assay in Male Balb/c nude mice, nanoparticles significantly inhibited tumor growth after laser irradiation, and the tumor volume remained almost constant for 21 days.

The therapeutic effectiveness of traditional photosensitizer-loaded drug delivery systems is severely limited by the photosensitizer aggregation quenching effect. Zhao et al. [38] designed novel heterodimeric prodrugs (PR104A-S-PPa and PR104A-TK-PPa) by linking PR104A, which is sensitive to hypoxia, and pyropheophorbide a (PPa) via TK bonds. Under light irradiation, PPa reacts with sulfur-containing bonds to free radical cations via photoinduced electron transfer, leading to the breakage of the C-S bond and the release of PR104A, followed by the decomposition of the nanoparticles. The excited PPa then reacts with the surrounding O_2_ to generate ROS, which kills tumor cells and induces the cleavage of the TK bond, further releasing PR104A. Compared with that of free PR104A, the blood circulation time of the prepared prodrug NPs was significantly longer, which could promote the accumulation of prodrug nanoparticles in tumors. In vivo antitumor studies in female BALB/c mice showed that after laser irradiation (660 nm, 200 mW/cm ^2^, 5 min) at two-day intervals over seven days, the tumor volume remained almost unchanged during the 12-day observation period.

In addition to cleaving chemical bonds, ROS can alter the hydrophobicity of unsaturated lipids by seizing hydrogen and peroxidation [73]. Yang et al. [39] encapsulated the photosensitizer chlorin e6 (Ce6) and the chemotherapeutic drug Pt (IV) in liposomes (Pt/Ce6-LP). Ce6 generates ROS under light irradiation (650 nm), and allyl hydrogens in the liposome membrane layer reversibly form hydrophilic lipid peroxides in the presence of ROS. This changes the hydrophobicity of the liposome membrane; thus, releasing Pt (IV) from the liposomes. In the absence of light irradiation, approximately 30 % of the Pt (IV) was released after 24 h. However, under light irradiation (650 nm, 0.5 W/cm ^2^, 3 min), Pt/Ce6-LP released Pt (IV) quickly, and approximately 60 % of the Pt (IV) was released within 24 h after the two irradiations. In the apoptosis assay, a better apoptosis rate was observed in the light-irradiated Pt/Ce6-LP group than in the non-light-irradiated group, showing good photocontrol. In the BALB/c nude mouse model, after three injections and six light irradiations (650 nm, 0.5 W/cm ^2^, 10 min), the tumor growth was significantly inhibited within 13 days.

The process of ROS generation accompanied by oxygen consumption creates a microenvironment conducive to the occurrence of reduction reactions in which the reactive groups are easily reduced [74]. Ma et al. [40] prepared tumor delivery nanoparticles (^DA^NP _VP&DOX_) from polyethylene glycol-polyphosphate (PEG-b-P (AEP-g-DA/NI)) for tumor treatment. Under X-ray irradiation, verteporfin (VP) in ^DA^NP _VP&DOX_ converted O_2_ to cytotoxic ^1^O_2_, causing cancer cell apoptosis. Moreover, nitroimidazole (NI) in polyphospholipids is converted to aminoimidazole under hypoxic conditions, which leads to a change in the hydrophobicity of nanoparticle shells and nanoparticle degradation. The experimental results showed that the amount of DOX released was directly proportional to the intensity of the X-rays and was significantly greater than that of the release from the ^DA^NP _VP&DOX_ under dark conditions. In addition, the release of DOX from the ^DA^NP _VP&DOX_ showed a pulse-controllable pattern with the switching cycle of X-rays, and about 50 % of the DOX could be released by irradiation 4 times in 7 h. The release of DOX slowed after the irradiation stopped, indicating good light-controlled release of the drug. In the in vivo anti-tumor assay, mice were injected intravenously every five days and irradiated with X-rays (4Gy, 10 min) 24 h after the injection of the drug. The results showed that the tumor inhibition rate of the ^DA^NP _VP&DOX_ group was as high as 88.1 % within 14 days, which significantly inhibited the growth of tumors.

Rwei et al. [41] proposed a method for on-demand pain treatment using ROS-sensitive liposomes for multiple, repeatable, and adjustable releases of tetrodotoxin (TTX) and dexmedetomidine (DMED) under laser irradiation. In vitro drug release studies showed that in the absence of laser irradiation, a mixture of TTX-loaded liposomes and DMED-loaded liposomes released only 3 % of TTX during the first 3 h. Within 8 h, after three rounds of laser irradiation (730 nm, 100 mW/cm ^2^, 10 min), 20 %, 11 %, and 25 % of TTX were released, respectively. In vivo triggering experiments with adult male Sprague-Dawley rats showed that up to seven effective releases could be produced by laser irradiation (730 nm, 75 mW/cm ^2^,5 min), and that prolonged irradiation facilitated the release of a low dose of TTX even when the TTX was already released in bursts. In another study [42], liposomes loaded with TTX and a photosensitizer PdPC(OBu)_8_ under NIR laser irradiation produced ROS, causing peroxidation of liposome lipids to establish pores that allowed TTX release (Fig. 2c: Left). In vitro studies have shown that light irradiation (730 nm, 50 mW/cm ^2^, 10 min) can trigger the release of TTX from the liposomes, and this light-triggered TTX release can be repeated twice (Fig. 2c: Middle). In adult male Sprague-Dawley rats, laser irradiation (730 nm, 50 mW/cm ^2^, 15 min) triggered two effective pain thresholds (i.e., hind-paw thermal latency) increase at 16-h and 22-h time points after the injection of TTX-loaded liposomes (Fig. 2c: Right). In addition, the intensity and duration of anesthesia could be adjusted by the duration of irradiation and irradiance.

#### 2.1.3. Mechanism of PTE-triggered drug release

PTT involves converting light energy into heat energy using photothermal agents to increase the temperature of diseased tissues, thereby killing affected cells, this therapy plays a significant role in cancer treatment [75]. When a photothermal agent is combined with a temperature-sensitive carrier, the PTE of the photothermal agent will increase the local temperature of the carrier, triggering a phase change or increasing the permeability of the carrier, leading to drug release. Triggering drug release in this mode can combine PTT with chemotherapy to produce a synergistic effect, effectively killing and destroying diseased tissue or cells [76].

#### 2.1.4. PTE-triggered drug release systems

**Fig. 3.**
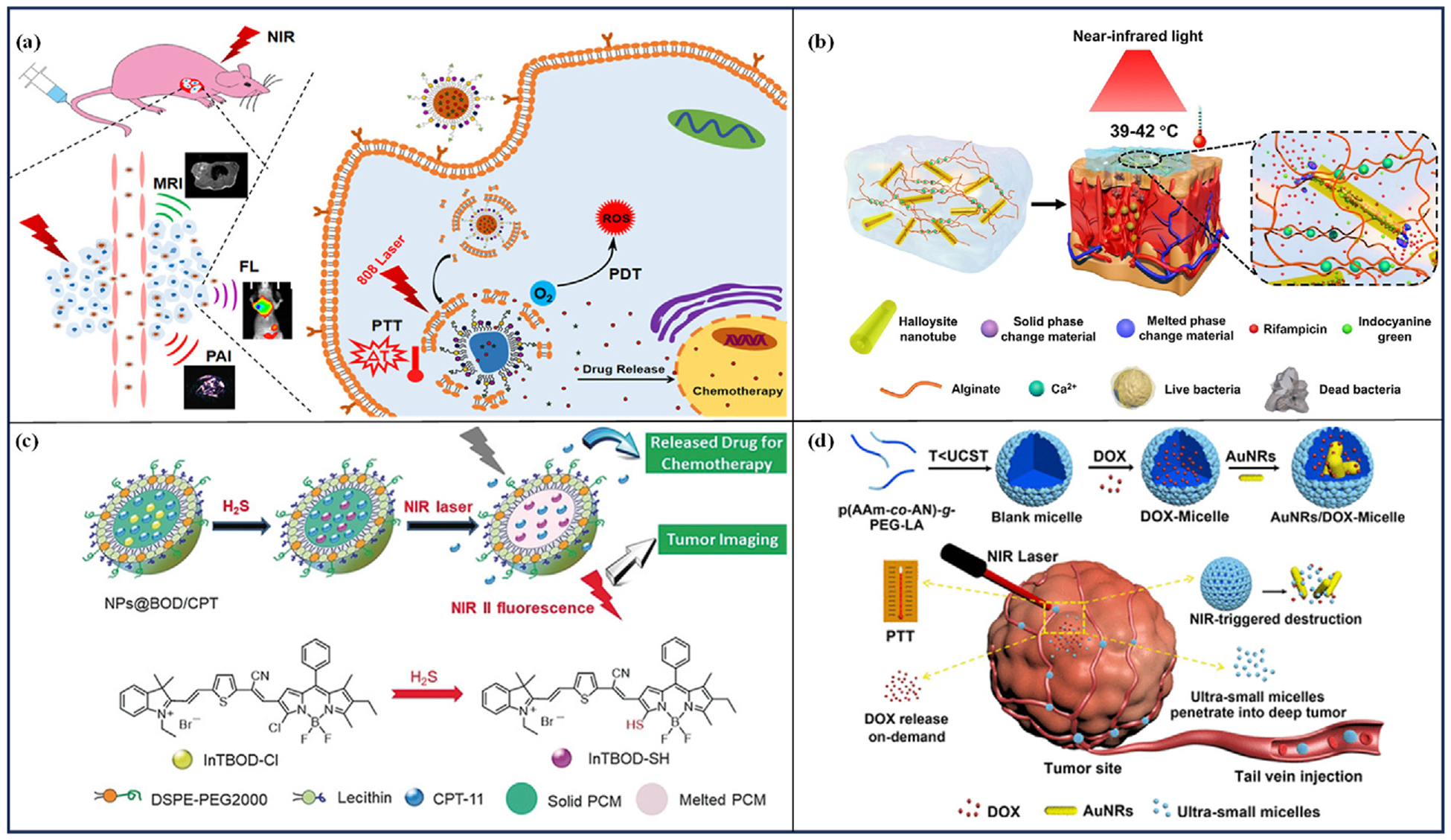
PTE triggered drug release: (a) Drug release of ID@TSL-Gd NPs in response to an NIR laser. Reprinted with permission from Ref. [43]. Copyright (2019) American Chemical Society. (b) Schematic illustration of the NIR-triggered release of drug molecules from nanotubes that were integrated with Alg hydrogel to promote infected wound healing. Reprinted with permission from Ref. [44]. Copyright (2022) Elsevier. (c) Schematic illustration of NP@BOD/CPT for on-demand CPT-11 release in light for cancer treatment. Reprinted with permission from Ref. [45]. Copyright (2019) John Wiley and Sons. (d) Schematic illustration of the size-shrinkable p (AAm-co-AN)-g-PEG-LA nanomicelles loaded with GNRs and DOX for anticancer applications. Reprinted with permission from Ref. [47]. Copyright (2021) American Chemical Society.

Dai et al. [43] prepared temperature-sensitive liposome nanoparticles (ID@TSL-Gd NPs) loaded with ICG/DOX and gadolinium (Gd) chelates by co-encapsulating lauric and stearic acids with ICG and DOX into liposomes and modifying the liposome surfaces with folic acid (FA) (Fig. 3a). Under NIR laser irradiation (808 nm, 0.5 W/cm ^2^, 10 min), the temperature of the solution was increased to above 45 °C within 5 min, at this temperature, the fatty acid crystals melt and trigger drug release. The prepared nanoparticles were irradiated twice within 100 min, and 55 % of the DOX was released. However, the nonirradiated nanoparticles did not release DOX, showing good trigger controllability. In vivo experiments showed that after injection of ID@TSL-Gd NPs into the tail vein of HeLa tumor-bearing nude mice, under NIR laser irradiation (808 nm, 0.5 W/cm ^2^, 10 min), the surface temperature of the tumors rapidly increased to 61 °C within 3 min, and the structure of liposomes was disrupted, resulting in the release of DOX. The volume of the tumors in the group treated with ID@TSL-Gd NPs was progressively reduced over a 14-day observation period. Furthermore, FA facilitated targeted and receptor-mediated drug delivery to mitigate the adverse effects of DOX.

Ye et al. [44] developed a NIR light-responsive hydrogel for wound healing by co-encapsulating fatty acids, rifampicin (RFP), and ICG into halloysite clay nanotubes, which were then mixed with Alg. Under NIR irradiation, ICG acts as a photothermal agent, converting light energy into heat, melting the fatty acids, and facilitating the release of RFP (Fig. 3b). In vitro experiments demonstrated that after 12 rounds of NIR laser irradiation (808 nm, 3.0 W/cm ^2^, 6 min), 47 % of RFP was released within 144 min. In contrast, less than 5 % of the RFP was released without irradiation, and a burst release was observed during irradiation. Antimicrobial testing in vitro revealed that co-culturing the hydrogel with *Staphylococcus aureus* suspension resulted in a nearly 100 % mortality rate of *Staphylococcus aureus* after three rounds of laser irradiation (808 nm, 3.0 W/cm ^2^, 30 s). In vivo antimicrobial studies indicated that following the implantation of the hydrogel and five rounds of laser irradiation (808 nm, 1.5–2.0 W/cm ^2^, 7 min), there was no pus at the wound site in rats, *Staphylococcus aureus* was completely eradicated within 7 days, and the wound had fully regenerated after 21 days.

Shi et al. [45] prepared thermoresponsive nanoparticles (NPs@BOD/CPT) using lauric acid and stearic acid, incorporating boron-dipyrromethene (InTBOD-Cl) and camptothecin-11 (CPT-11) as guest molecules (Fig. 3c). Activation of InTBOD-Cl can be triggered by H_2_S produced by cancer cells, upon NIR laser irradiation, the activated InTBOD-Cl can raise the temperature of the nanoparticle solution to 39 °C within 5 min, leading to a phase transition that triggers the release of CPT-11 for the treatment of colorectal cancer. Following laser irradiation (785 nm, 5.12 W/cm ^2^, 10 min), the temperature of the HCT116 tumors injected with nanoparticles gradually increased to 44.9 °C. This value was greater than the eutectic matrix melting point, releasing encapsulated CPT-11. Cell viability assays demonstrated a notable decline in cell viability after light exposure compared to the control, indicating effective photo-controllability of drug release. Intratumoral injection of nanoparticles into HCT116 tumor-bearing mice and laser irradiation (785 nm, 1 W/cm ^2^, 10 min) after 10 min showed inhibition of tumor growth over a 14-day monitoring period.

Clinically, the main method of abscess treatment is systemic high-dose antibiotic therapy followed by incision and drainage [77,78], but the treatment process is very painful due to the antibiotic-resistant bacteria, which requires multiple incisions, drainage, and wound tamponade. Gao [46] et al. prepared a NIR light-triggered thermo-sensitive hydrogel drug reservoir by mixing polydopamine (PDA) nanoparticles containing ciprofloxacin (Cip, a potent antibiotic) with glycol chitosan (GC) to form an injectable hydrogel (Gel-Cip). Under the irradiation of NIR light, the Cip in Gel-Cip was rapidly released, while the light energy was converted into heat energy due to the PTE of PDA nanoparticles, which would generate a localized high temperature. The two act synergistically to kill the surrounding bacteria. The bactericidal efficiency of Gel-Cip was as high as 98.9 % in S. aureus-infected mice irradiated by NIR laser (808 nm, 0.5 W/cm ^2^, 10 min), and the PTE of Gel-Cip was also demonstrated, with the temperature of bacterial infected area rising to 46.1 °C after irradiation. The combined effect of the two showed good therapeutic efficacy, and the wounds of mice in the Gel-Cip + NIR treatment group almost disappeared on day 4, with only 6.4 % of the wound area remaining.

Besides organic photothermal agents, inorganic counterparts like GNR, oxide nanomaterials, black phosphorus, and graphene oxide have also been employed to induce drug release [9,79,80]. In contrast to organic photothermal agents, inorganic ones offer higher photothermal conversion efficiency and a simpler synthesis process [81]. Wu et al. [47] developed a novel NIR laser-triggered size-contractable nanocolloid by incorporating GNR into polymeric micelles composed of the block copolymer polyacrylamide-acrylonitrile-polyethylene glycol-lipoic acid (p (AAm-co-AN)-g-PEG-LA) (Fig. 3d). The nanocolloid has an upper critical solution. temperature (UCST), and when the NIR laser irradiates the tumor site, the GNRs convert the light energy into heat, causing the large-sized nanocolloid to break up into ultra-small micelles (7 nm), which penetrates the tumor and releases the DOX. In the absence of light irradiation, 32 % of the drug was released within 6 h. However, following 4 NIR laser irradiation (808 nm, 2 W/cm ^2^, 5 min) sessions about 78 % of the DOX was released within 6 h. The anti-tumor efficacy of nanocolloid was studied in HepG2 xenograft tumor-bearing mice. The tumor was significantly inhibited after being irradiated with NIR light (808 nm, 2 W/cm ^2^, 2 min) with a tumor inhibition rate of 93 %.

**Fig. 4.**
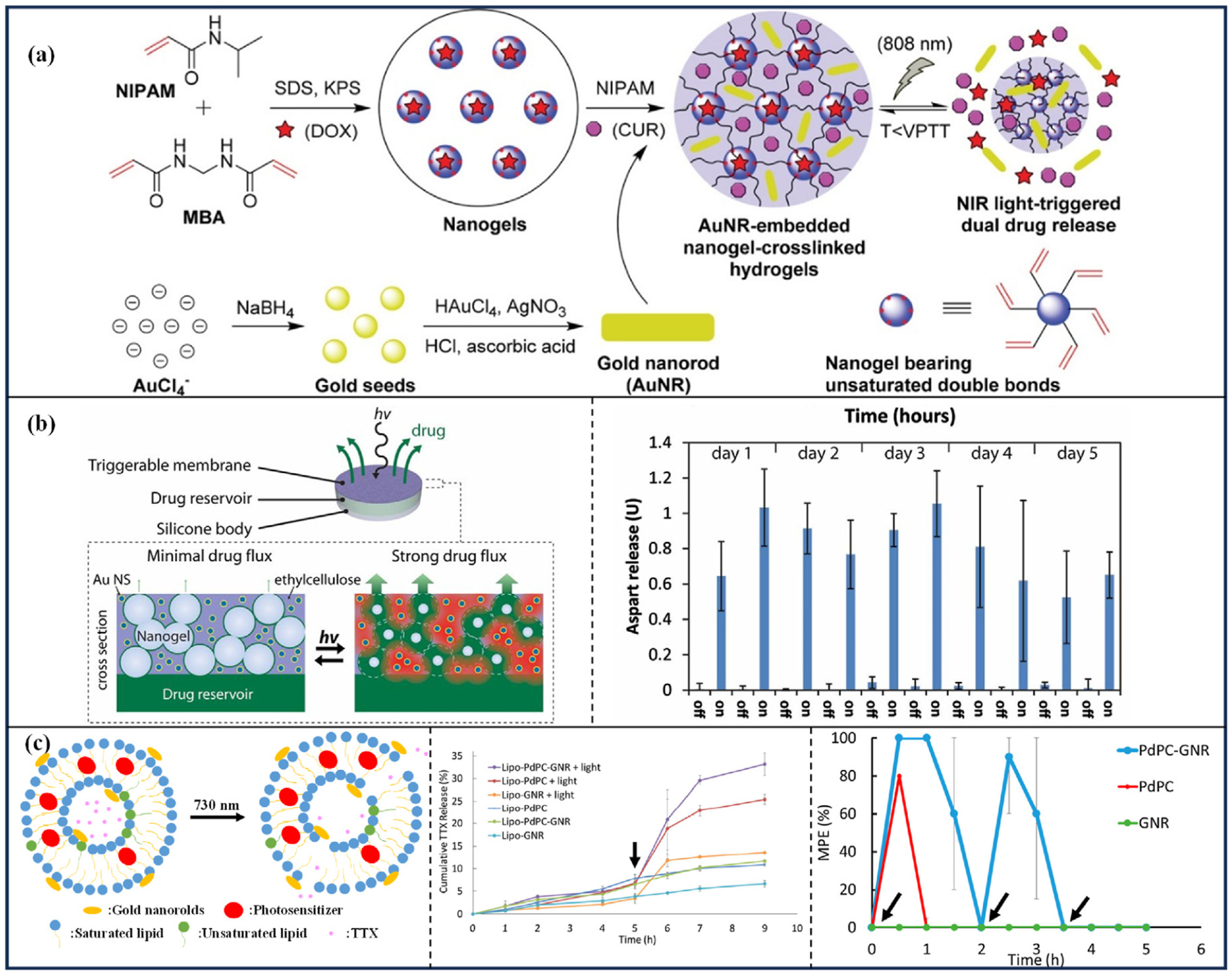
Drug release triggered by the PTE of gold nanoparticles: (a) Schematic illustration of prparation of the GNRs, nanogels, and nanogel-crosslinked hydrogels. The local heating induced by NIR light (808 nm) resulted in the contraction of the hydrogels and therefore on-demand drug release. Reprinted with permission from Ref. [48]. Copyright (2019) John Wiley and Sons. (b) Left: Schematic illustration of light triggering the release of a drug (aspartic acid) for the treatment of diabetes. Right: Devices incorporating aspartic acid can be activated for up to 10 effective releases over a span of 5 days. Reprinted with permission from Ref. [51]. Copyright (2014), National Academy of Sciences. (c) Left: Under light, GNR and PDPC synergistically triggered TTX release for the treatment of pain. Middle: In vitro release profile of TTX after a single laser irradiation (730 nm, 55 mW/cm ^**2**^, 3 min) within 9 h. Lipo + PdPc + GNR + Light group showed the highest release rate, about 33.5 %. Right: NIR laser irradiation (730 nm, 200 mW/cm ^**2**^, 3 min) induced liposomes (PdPc-GNR) encapsulating TTX to produce anesthesia twice in rat footpads. Reprinted with permission from Ref. [52]. Copyright (2017), American Chemical Society.

Jiang et al. [48] prepared a NIR light-triggered nanogel-crosslinked hydrogel dual-release system by N-isopropylacrylamide (NIPAM) precipitation polymerization using nanogel as a crosslinking agent (Fig. 4a). DOX was encapsulated in the nanogel and curcumin (CUR) and GNR were encapsulated in the hydrogel. Irradiation of GNR produces a PTE, which generates a localized phase transition in the nanogel, altering the crosslinked structure of the gel and releasing the encapsulated drug. After 10 irradiations (808 nm, 650 mW/cm ^2^, 7 min), the release of DOX and CUR reached more than 60 % in 70 min, whereas in the absence of irradiation, it took 48 h for CUR to reach 60 %, and the cumulative release of DOX was only about 55 % in 120 h.

Shagan and collaborators [49] prepared a NIR-responsive composite material by blending star-shaped PEG and poly (ε-caprolactone) (PCL) with gold nanoshells (GNSs) and evaluated the responsive release of the system using CPT as a guest molecule. During CPT release, exposure to NIR light (808 nm) for 5 min every hour led to a release of CPT reaching 40 % within 4 h, and burst release was observed. In contrast, the CPT release from unirradiated samples was about 5 %. A similar trend was observed with bupivacaine hydrochloride (BUPI-HCl), which displayed a stepwise release profile. The release of BUPI-HCl reached 50 % after three irradiations within 6 h. Without irradiation, less than 30 % of BUPI-HCl was released from the composite.

Zhan et al. [50] modified temperature-sensitive liposomes with GNRs, and under NIR laser irradiation, the PTE of the GNRs caused the liposomes to undergo a phase transition, which resulted in the release of encapsulated TTX and DMED for the treatment of pain. In vitro drug release studies showed that the temperature of the liposomes could rise above the phase transition temperature under NIR laser (808 nm, 75 mW/cm ^2^, 15 min) irradiation, and the liposomes were able to maintain the intact structure after up to six cycles. The drug-loaded liposomes produced a small amount of burst release after injection into the footpad of rats, but the drug could still be triggered by the laser (808 nm, 75, 141, and 272 mW/cm ^2^ for 10 min) four times within four days after the burst release to produce effective anesthesia. Duration and intensity of anesthesia were positively correlated with the laser intensity.

Brian et al. [51] developed an innovative implantable drug reservoir specifically designed for the delivery of insulin (INS) analogs to treat diabetes (Fig. 4b: Left). This device incorporates a nanocomposite membrane embedded with hollow gold nanoshells (AuNS). Upon exposure to NIR light, AuNS convert light energy into heat, elevating the temperature of the membrane. This temperature increase induces the collapse of the polymer nanogel network within the membrane, thereby facilitating the release of the encapsulated drug. In vitro studies demonstrate that with NIR laser irradiation (808 nm, 570 mW/cm ^2^), the complete release of the aspartic acid is achieved within a minimum of 3 h. When subjected to bi-daily laser exposures (808 nm, 570 mW/cm ^2^, 30 min), the device can release aspartic acid for up to five days, with aspartic acid release becoming nearly negligible after cessation of laser irradiation (Fig. 4b: Right). Upon subcutaneous implantation in diabetic Sprague-Dawley rats, the device effectively triggers aspartic acid release in vivo upon NIR light exposure. The therapeutic efficacy of the device is positively correlated with the laser power, and the drug reservoir continues to perform effectively, delivering the aspartic acid even on the 14th day following three triggering sessions.

Rwei et al. [52] proposed the use of liposomes loaded with TTX and photosensitizer 1,4,8,11, 15, 18,22,25-octabutoxyphthalocyaninato-palladium (II) (PdPC) and modified their surface with GNR. The ROS generated by the photosensitizer under light exposure altered the permeability of the liposome membrane, and the PTE of the GNR caused the liposomes to undergo a phase transition, and the two acted synergistically to trigger the release of TTX (Fig. 4c: Left). In vitro experiments on drug release showed that GNR and PdPC are complementary in power, at low power irradiation (730 nm, 35 mW/cm ^2^, 5 min), the PDE of PdPC makes the main driver of drug release, and at higher power (730 nm, 200 mW/cm ^2^, 5 min), the PTE of GNR is the main driver of drug release. With light irradiation at the fifth hour (730 nm, 55 mW/cm ^2^, 3 min), approximately 33.5 % of the TTX was released within 9 h, whereas only 11.5 % of the TTX was released when not irradiated by the laser (Fig. 4c: Middle). In vivo experiments, TTX-containing liposomes were injected into rat footpads, the drug release could be triggered twice consecutively under laser (730 nm, 200 mW/cm ^2^, 3 min) irradiation to produce anesthesia (Fig. 4c: Right).

Kim et al. [53] fabricated gel beads (p (NIPAM-co-VP)/MNPs) composed of temperature-responsive poly (N-isopropylacrylamide-vinyl-2-pyrrolidone (p (NIPAM-co-VP) hydrogels and magnetite nanoparticles (MNPs). The MNPs dispersed in the hydrogel matrix absorbed Vis light and generated heat, and the volume of the gel beads rapidly contracted when the temperature rose above the lower critical solution temperature (LCST), thus promoting drug release. Upon cessation of irradiation, the hydrogel absorbed water from the medium and returned to its initial state, thus preventing the release of dexamethasone (DEX) from the gel beads. The experimental results showed that DEX was released rapidly during light irradiation (blue light, 474 mW/cm ^2^, 1 h) with a release rate as high as 10.2 % h ^−1^, and the release rate decreased to 1 % h ^−1^ after the cessation of light irradiation, which exhibited good phototriggered properties. In addition, the light-triggered release rate was proportional to the light intensity, which increased from 6.5 % h ^−1^ to 9.1 % h ^−1^ when the light intensity was increased from 47.5 mW/cm ^2^–353.7 mW/cm ^2^.

Qiu et al. [54] prepared a black phosphorus (BP) hydrogel (BP@Hydrogel) by blending agarose and polyethylene glycolized black phosphorus nanosheets (BPNS) at 60 °C. BP transformed light into heat, leading to the softening and melting of hydrogel nanostructures, facilitating drug release. The photothermal efficiency of BPNS was evaluated using an 808 nm NIR laser at a power density of 1.0 W/cm ^2^, achieving a photothermal conversion efficiency of up to 38.8 %. The rate of drug release could be finely tuned by adjusting various factors, including light intensity, exposure duration, BP concentration, and the composition of the hydrogel. Studies in tumor-bearing nude mice showed that the DOX-loaded BP@Hydrogel effectively ablated tumors under light irradiation (808 nm, 1.0 W/cm ^2^, 5 min), with no tumor regrowth observed after 14 days. Meanwhile, the body weight of nude mice was not significantly affected, suggesting the absence of acute side effects, and both the agarose hydrogel and the BPNS were degradable upon completion of treatment, making them promise for clinical translation.

Nanographene oxide (nGO) demonstrates high photothermal conversion efficiency when exposed to NIR light and shows significant potential for cancer therapy [82–84]. Sahu et al. [55] utilized embedded nGO as a photosensitive switch in thermosensitive liposomes, controlling drug release through NIR light. Calcein was employed as a model drug to investigate light-triggered release. Both femtosecond pulsed laser irradiation (805 nm) and continuous-wave laser irradiation (808 nm, 1 W/cm ^2^, 10 min) successfully stimulated the release of the dye, enabling up to four distinct release cycles. Upon cessation of irradiation, the release rate of calcein from the nGO-containing liposomes decreased markedly, this reversible “on-off” release pattern suggests that the laser exposure did not damage the integrated nGO liposomes.

#### 2.1.5. Mechanism of photocleavage-triggered drug release

Photocleavage-triggered drug release typically entails the drug molecule being bound to a carrier via a photosensitive bond through a linker (such as a coumarin derivative or S, S-tetrazine), in a state where the drug remains inactive. Upon light irradiation, the photosensitive bond is broken, resulting in disruption of the structure of the carrier and release of the drug [85].

#### 2.1.6. Photocleavage-triggered drug release systems

**Fig. 5.**
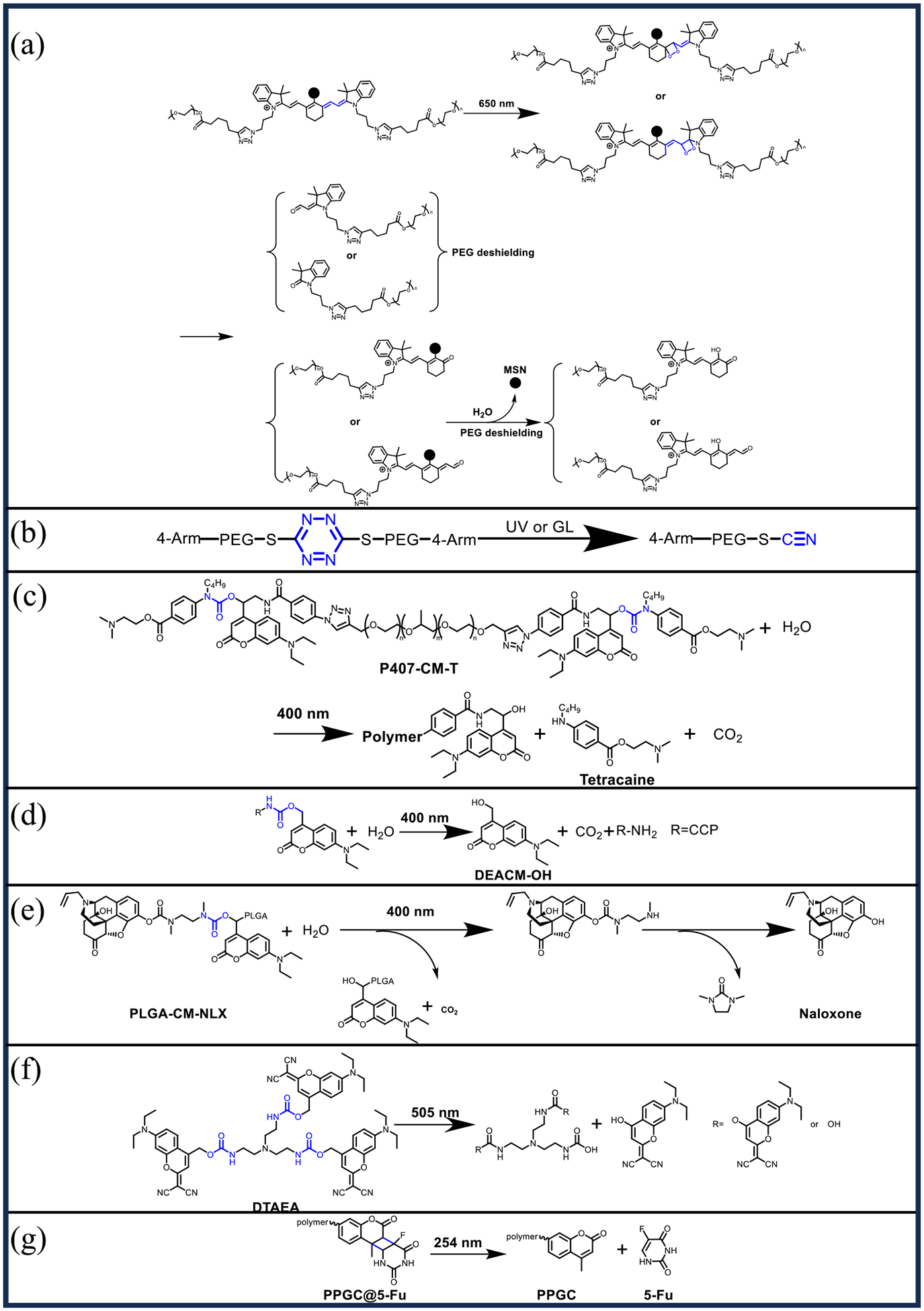
Summary of photo-induced cleavage of photosensitive bonds: (a) Photo-oxidative breaking and hydrolysis of Cy polyene bonds under light dissociates PEG from the MSN surface. (b) The S, S-tetrazine chromophore decomposes under UV or GL irradiation, resulting in hydrogel degradation. (c) Light-triggered cleavage of the carbamate bond in P407-CM-T releases tetracaine. (d) Light-triggered cleavage of the carbamate bond releasing CPPs. (e) Light-triggered carbamate bond cleavage resulting in delta molecule decomposition. (f) Light-triggered decomposition of delta molecule. (g) Release of 5-FU triggered by retro-cycloaddition reaction under 254 nm UV irradiation.

Li et al. [56] presented photo-induced PEG deshielded mesoporous silica nanocarriers (SiO_2_@DOX-Cy-PEG) for on-demand drug release in acidic tumor microenvironments. The photosensitizer heptamethyl cyanine dye (Cy) was utilized as a linker to attach PEG to the surface of the nanoparticles. Upon exposure to NIR light at 650 nm, Cy underwent photo-dissociation, resulting in the detachment of PEG from the silica nanoparticles and subsequent release of the encapsulated DOX (Fig. 5a). The system demonstrated significant pH responsiveness, maintaining high stability with minimal drug leakage at physiological pH 7.4, where less than 5 % of DOX was released over 60 h. In contrast, at acidic pH 4.5, the release rate of DOX increased to 40.7 % after 60 h. The effectiveness of light in facilitating drug release was validated through apoptosis assays and xenograft 4T1 tumor models in BALB/c mice. Following light exposure, the release rate of DOX increased to 71.2 %, light irradiation (650 nm, 0.2 W/cm ^2^, 30 min) significantly reduced the survival rate of 4T1 and HeLa cells. The tumor volume of the SiO_2_@DOX-Cy-PEG-treated group was comparable to that of the free DOX group throughout the 20-day observation period. During the 20-day observation period, tumor growth in the SiO_2_@DOX-Cy-PEG treated group was inhibited, with tumor volume comparable to that of the free DOX group.

Tetrazine-based chromophores are utilized as photochemical triggers due to their rapid response to light stimuli and their non-toxic photoreaction products [86–89]. Wang et al. [57] reported the development of a novel photocleavable hydrogel incorporating 3,6-dichloro-1,2,4,5-tetrazine as the cross-linking linker, which links a 4-armed thiol-capped PEG through S, S-tetrazine. Under UV or green light (GL) irradiation, the hydrogel undergoes cleavage because the S, S-tetrazine bonds are broken, thereby releasing the encapsulated drug (Fig. 5b). The photo-cleavage of the S, S-tetrazine cross-linking unit yields only inert PEG analogs and nitrogen, minimizing toxicity to biological tissues. In experiments using DOX as a model drug, the drug release rate under UV illumination was found to be proportional to the UV light’s power density. Due to the limited penetration and phototoxicity associated with UV light, the use of GL for inducing drug release was explored. Results demonstrated that the drug release rate under GL increased proportionally with the power density of the light. In vivo experiments with GL irradiation of nude mice with subcutaneous MDA-MB-231 tumors effectively triggered the release of the dye Cyanine 5.5 from the injected gel. Furthermore, DOX-loaded gels subjected to GL irradiation (532 nm, 1.12 W/cm ^2^, 5 min, once a day for five days) inhibited tumor growth and significantly reduced tumor size in the mice. This research provides a promising and practical approach for creating Vis-light cleavable hydrogels with tunable and on-demand drug release capabilities.

Coumarin exhibits the capability to absorb UV light and subsequently emit light at lower wavelengths [90]. Prodrugs that utilize coumarin as a linker and connect drug molecules via carbamate bonds remain stable under physiological conditions, but these carbamate bonds can be cleaved by light, leading to the release of the drug molecules. Zhang et al. [58] developed a photo-triggered polymer-drug conjugate, P407-CM-T, which combines a tetracaine prodrug (CM-T) with Poloxamer 407. In vitro experiments demonstrated that blue light irradiation (400 nm, 50 mW/cm ^2^, 5 min) effectively disrupted the carbamate bonds, releasing the tetracaine molecule (Fig. 5c). In vivo studies involved injecting the conjugate into the footpads of rats. Rats not exposed to irradiation did not experience local anesthesia, whereas those exposed to blue light (400 nm, 200 mW/cm ^2^, 2 min) achieved local anesthesia. Moreover, increasing the light power to 300 mW (400 nm, 300 mW/cm ^2^, 2 min) resulted in up to five drug releases. The duration of anesthesia was positively correlated with both the power and duration of blue light irradiation, demonstrated a linear relationship with the irradiation energy density. This indicates that the therapeutic effect can be finely tuned by adjusting the light intensity and duration, allowing for customizable treatment in response to varying patient needs.

Wang et al. [59] introduced a novel strategy for light-induced targeted therapy of choroidal neovascularization using polyethylene glycol-polylactic acid (PEG-PLA) polymer chain-modified cell-penetrating peptides (CPPs) to deliver DOX. In this approach, CPPs were covalently linked to 7-(diethylamino)-4-(hydroxymethyl)-coumarin (DEACM) through a carbamate linker, which inactivated the CPPs and then bound them to the PEG-PLA chain. Upon exposure to blue light, the carbamate bond was cleaved, releasing DEACM and reactivating the CPPs (Fig. 5d). This reactivation caused the nanoparticles to aggregate at the irradiation site, thereby enhancing the therapeutic efficacy of the encapsulated DOX. In vitro cellular experiments demonstrated that the cellular uptake rates of the blue-light (400 nm, 50 mW/cm ^2^, 1min) irradiated nanoparticles and those with unmodified CPPs were comparable and significantly higher than those of the non-irradiated group, indicating that laser stimulation effectively activated the CPPs. In animal studies, laser irradiation (400 nm, 50 mW/cm ^2^, 30 s) of the rat’s eyes immediately after intravenous injection, followed by euthanasia within 24 h, revealed that the laser-irradiated group had the highest accumulation of nanoparticles in the ocular tissues.

Zhang et al. [60] employed a similar strategy to develop a photo-responsive polymer-naloxone conjugate aimed at mitigating morphine overdose. In this approach, naloxone was covalently linked to the polymer Poly (lactic-co-glycolic) acid (PLGA) through a photocleavable coumarin linker to create injectable nanoparticles. Upon exposure to light (400 nm, 300 mW/cm ^2^, 2 min), the carbamate bond was cleaved, releasing naloxone (Fig. 5e). Following subcutaneous injection into the dorsal region of mice, drug release could be precisely triggered by light irradiation (400 nm, 300 mW/cm ^2^, 2 min). During the 20-day observation period, the drug could be triggered for release up to four times, with significant changes observed in the mice’s thermal latency each time, demonstrating good reproducibility. Furthermore, the drug delivery system exhibited high stability, remaining effective in vivo for up to one month and capable of triggering naloxone release even on the thirtieth day.

**Fig. 6.**
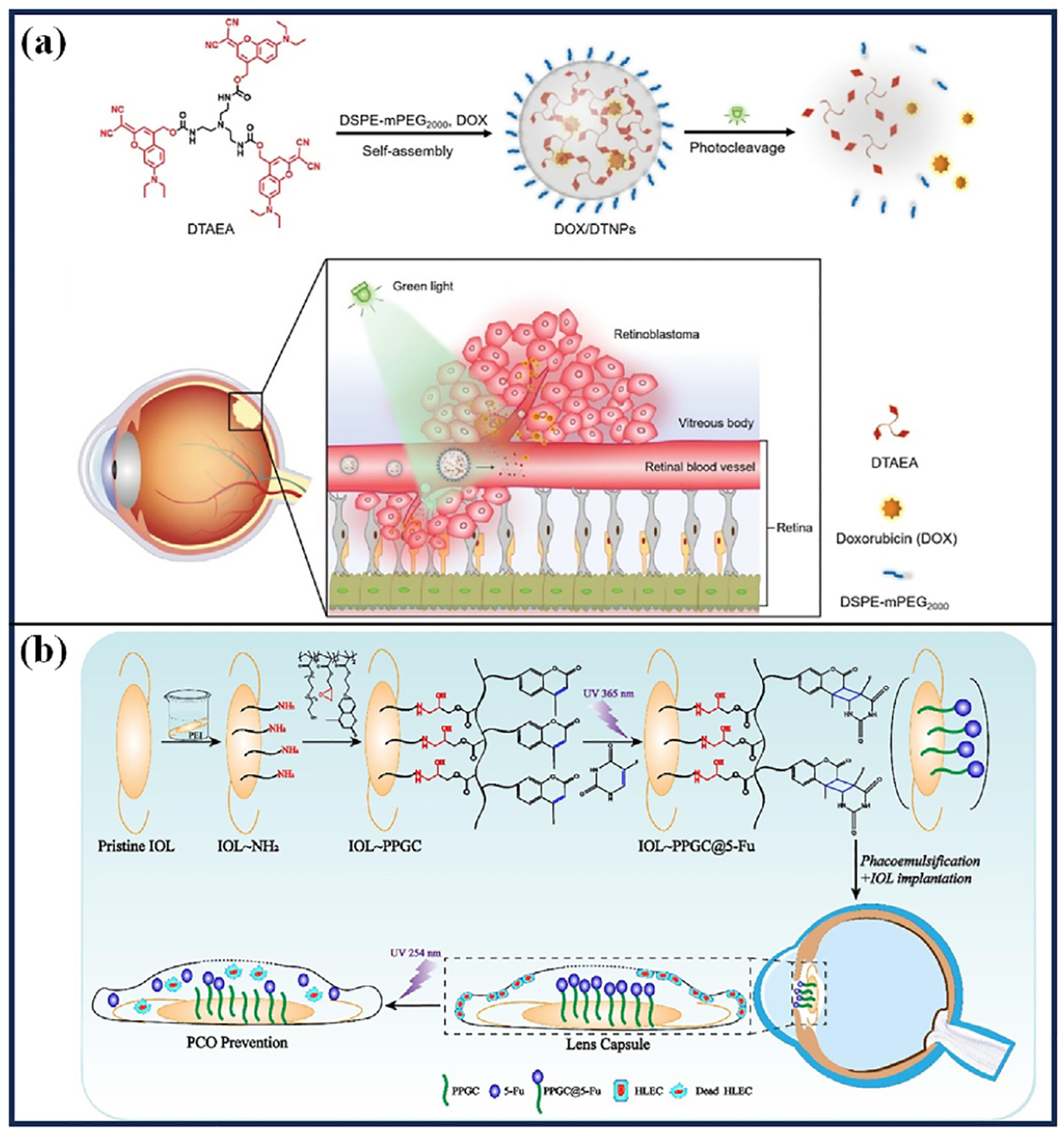
Photocleavage-triggered drug release: (a) Schematic illustration of light-exposed carbamate bond breakage and carrier disruption to release DOX for retinoblastoma treatment. Reprinted with permission from Ref. [61]. Copyright (2021) John Wiley and Sons. (b) Schematic illustration of hydrophilic and photo-responsive drug release coating construction on the IOL surface as well as the photo-controlled posterior capsular opacification prevention of the functionalized IOL after intraocular implantation. Reprinted with permission from Ref. [62]. Copyright (2021) Elsevier.

Long et al. [61] synthesized the delta molecule DTAEA by coupling a GL-responsive dicyanomethylene coumarin (DEAdcCM) with tris(2-aminoethyl) amine (TAEA). This was then coassembled with DSPE-mPEG 2000 to form DSPE-mPEG 2000/DTAEA nanoparticles (DTNPs) for the treatment of retinoblastoma (Fig. 6a). After intravenous injection of DOX-loaded nanocarriers (DOX/DTNPs), GL at a wavelength of 505 nm triggered the decomposition of the delta molecules (Fig. 5f), leading to the disintegration of the nanocarriers within the retinal vasculature. This caused the release of the intraocular drug and effectively inhibited the growth of retinoblastoma. Light-triggered drug release in tumor-bearing mice after intravenous injection of DOX/DTNPs showed that intraocular drug release was achieved in the light-irradiated eye. In contrast, drug release was not observed at other sites, indicating that ocular irradiation released the drug only in the eye and did not produce systemic toxicity. Following five injections over 12 days, coupled with light irradiation (505 nm, 50 mW/cm ^2^, 5 min), DOX/DTNPs exhibited a significant inhibitory effect on the tumors, which were nearly completely eradicated by day 25. There was no significant change in the body weights of the mice during the treatment period, which demonstrated a good safety profile.

Xia et al. [62] developed a novel approach for treating posterior capsule opacification (PCO) following cataract surgery by utilizing a coumarin methacrylate copolymer (PPGC) as a linker to attach 5-fluorouracil (5-FU) to the surface of an intraocular lens (IOL) (Fig. 6b), forming a hydrophilic, photo-controlled drug release coating on the IOL. UV irradiation at 365 nm, a [2 + 2] cycloaddition reaction between coumarin and 5-FU occurs, which immobilizes 5-FU on the IOL surface. This coating allows for controlled drug release in response to UV light at 254 nm (Fig. 5g). In vitro studies demonstrated that the PPGC@5-FU aqueous solution could rapidly release 5-FU under 254 nm UV light. Following the cessation of light irradiation, the drug release rate slowed, with the amount released being dependent on the intensity and duration of the irradiation. In vivo testing with IOL-PPGC@5-FU implanted in the eyes of Japanese white rabbits, followed by three UV irradiations (254 nm, 4.5 mW/cm ^2^, 5 min), demonstrated promising results. After one month, only a minimal number of cells were observed in the outermost layer of the IOL, and the optical zone remained clear with no significant cell proliferation throughout the posterior capsule. These findings indicate that the hydrophilic, photo-controlled drug coating on the modified IOL effectively inhibits the progression of PCO.

#### 2.1.7. Mechanism of light-triggered drug release based on UCNPs

NIR light penetrates tissues more deeply and exhibits lower tissue toxicity compared to UV light [91]. However, breaking chemical bonds with NIR light is more challenging than with UV light [92]. An alternative approach for NIR light-triggered drug release involves UCNPs. UCNPs can convert long-wavelength NIR absorption into short-wavelength emission through a nonlinear optical process [93], facilitating bond cleavage and enabling controlled drug release [94,95].

#### 2.1.8. Light-triggered drug release systems based on UCNPs

**Fig. 7.**
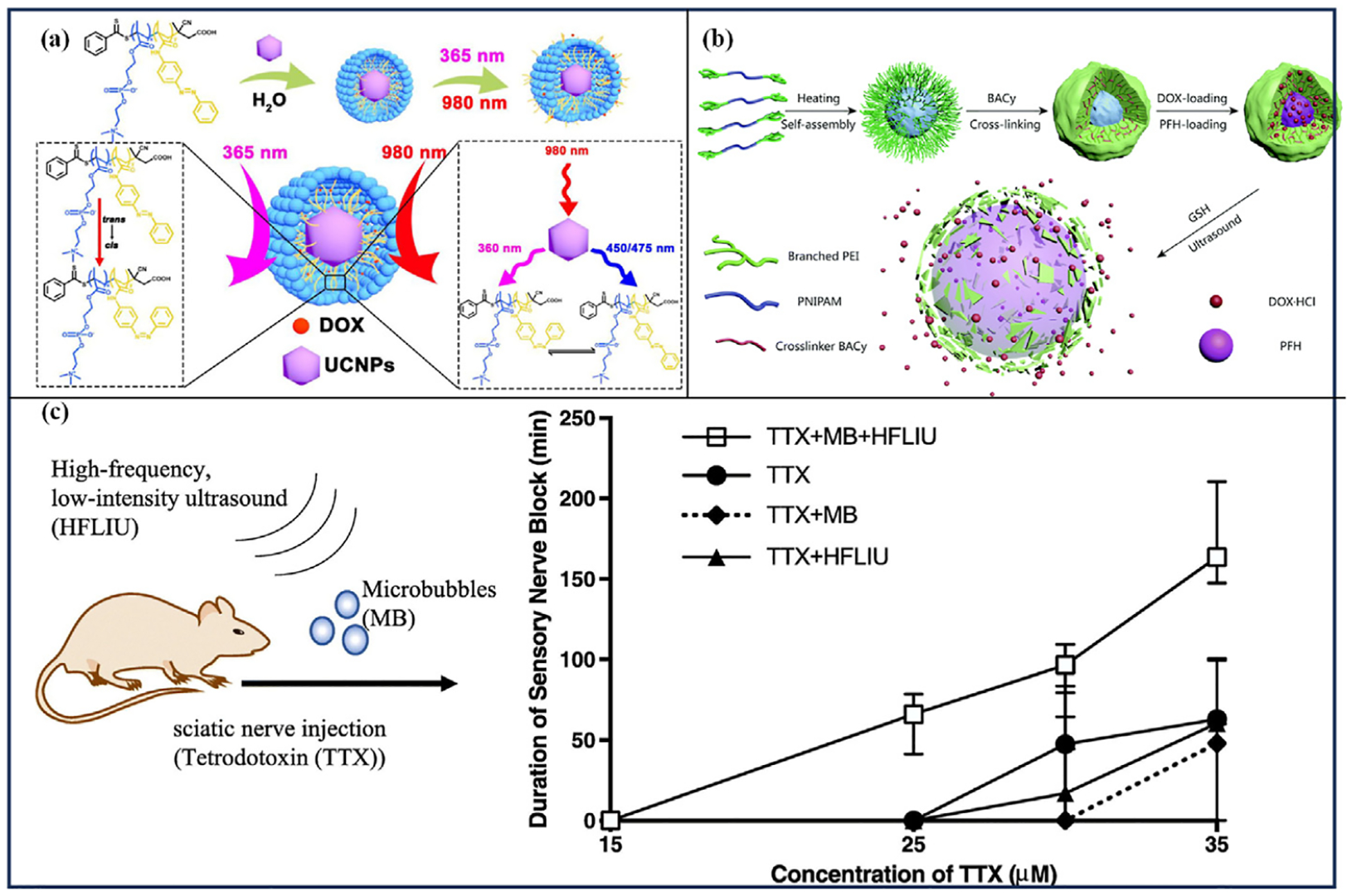
Light-triggered drug release systems based on UCNPs: (a) Schematic illustration of the preparation of the UCNPs@PMA micelles and DOX release. Reprinted with permission from Ref. [64]. Copyright (2022) American Chemical Society; Cavitation effect-triggered drug release: (b) Schematic illustration of nanogel preparation and dual stimulation triggering drug release. Reprinted with permission from Ref. [105]. Copyright (2017) Royal Society of Chemistry. (c) Schematic illustration of HFLIU combined with microbubbles promotes the release of TTX and enhances the efficacy of nerve blockade. Reprinted with permission from Ref. [109]. Copyright (2018) Elsevier.

Ma et al. [64] synthesized a UV-sensitive poly (2-methacryloyloxyethylphosphorylcholine-azobenzylmethacrylamide) (PMA) amphiphilic copolymer using reversible addition. They then encapsulated UCNPs within PMA micelles to create dual-responsive micelles (UCNPs@PMA) that respond to both UV and NIR light (Fig. 7a). When exposed to NIR light, the UCNPs embedded in the PMA micelles convert NIR excitation into UV/Vis emitted light. This process induces a reversible isomerization of the azo group and causes continuous rotational-reversal movement of the micelle core, inducing drug release. In vitro experiments showed that at pH 5.5, the cumulative release rate of DOX from UCNPs@PMA micelles was only 37 % over 48 h. However, this rate increased to approximately 81 % after 10 min of NIR radiation (980 nm, 1.00 W/cm ^2^, 10 min). At a neutral pH of 7.4, the pH-responsive release rate of DOX was lower compared to the acidic environment, suggesting that the UCNPs@PMA micelles possess effective tumor-targeting properties. In cytotoxicity studies, the viability of L929 and HeLa cells treated with DOX-loaded UCNPs@PMA micelles significantly decreased under NIR laser irradiation (980 nm, 0.75 W/cm ^2^, 10 min), indicating that NIR light effectively triggers the release of DOX.

Shi et al. [65] synthesized UCMS-S-BP nanoparticles by integrating UCNPs, mesoporous silica, and a photosensitive thioether linker (-S-BP). In this system, DOX was loaded into the mesopores of UCMS, and the -S-BP linker on the surface of the particles was bound to β-cyclodextrin (β-CD). This binding blocked the pores and prevented the release of the DOX. When exposed to ultra-low intensity NIR light (980 nm, 0.30 W/cm ^2^), the UCNPs convert NIR to UV light. This UV light cleaves the -S-BP linker, dissociates β-CD, and triggers the release of DOX. Drug release studies demonstrated that only 4.8 % of DOX was released in 120 min under no light conditions. However, DOX release increased significantly under NIR irradiation and was positively correlated with increasing power. Cytotoxicity experiments revealed a notable reduction in cell viability, from 52.2 % to 14.8 %, when the particle concentration was 500 μg/mL and the laser irradiation (980 nm, 0.3 mW/cm ^2^) time was extended from 5 to 20 min. These findings indicate that the UCMS-S-BP nanoparticles effectively leverage NIR light to control drug release.

Jiang et al. [66] developed nanoparticles with a core of UCNPs and surface-loaded with zeolitic imidazolate framework-8 and a NO donor, nitrosothiol. Under NIR light irradiation, UCNPs convert infrared light into UV light, which then cleaves the S-NO bonds in nitrosothiol, releasing NO for spinal cord injury repair. In vitro experiments demonstrated that, under NIR light irradiation (980 nm, 1.5 W/cm ^2^, 1 min), NO was rapidly released, resulting in significant improvements in cell differentiation rate and neurite length in the light-irradiated group, showing the potential to repair damaged neurons. In vivo experiments showed that, after irradiation (980 nm, 1.5 W/cm ^2^, 1 min), the nanoparticles significantly increased the axon length of zebrafish neurons by approximately 42 % compared to the untreated group and promoted motor recovery in rats with spinal cord injuries.

### 2.2. US-triggered drug release

US has been utilized for various applications including thermotherapy [96], imaging [97], and lithotripsy [98]. Fundamentally, US is defined as a periodically vibrating mechanical wave with frequencies exceeding 20 kHz [99]. US waves with frequencies ranging from 20 to 100 kHz are categorized as low-frequency ultrasound (LFUS) [100], whereas those with frequencies exceeding 1 MHz are classified as high-frequency ultrasound (HFUS) [101]. US has been widely praised for its safety, non-invasiveness, and high spatial resolution in biological applications [8].

In contrast to light-responsive systems, US exhibits high tissue penetration capability, with a penetration depth exceeding 10 cm in soft tissues [99]. US-triggered drug release is notably faster compared to other methods [102]. Additionally, US enhances tissue permeability and induces cellular phagocytosis of loaded therapeutic molecules or drugs, thereby enhancing drug efficacy [103]. Table 2 provides a summary of US-triggered drug release systems developed in recent years. Based on the different triggering principles, US-triggered drug release systems can be categorized into four categories: cavitation effect-triggered drug release systems, mechanical force-triggered drug release systems, sonodynamic effect (SDE)-triggered drug release systems, and thermal effect-triggered drug release systems.

#### 2.2.1. Mechanism of cavitation effect-triggered drug release

Cavitation occurs when microscopic bubbles within a liquid oscillate due to US, absorbing energy and expanding until they ultimately burst [122]. However, in drug delivery systems, the cavitation effect of the carrier itself may not be sufficient to trigger drug release. To enhance the responsiveness of carriers, phase change agents, such as perfluorocarbons (PFCs) and supercritical carbon dioxide, are often incorporated into the carriers. Upon exposure to US, these phase change agents vaporize, forming bubbles that subsequently burst. This burst generates shear forces that disrupt the carrier structure, facilitating drug release [123]. Additionally, the cavitation effect can increase cell membrane permeability, thereby accelerating drug absorption [124].

#### 2.2.2. Cavitation effect-triggered drug release systems

PFC are highly hydrophobic, chemically stable, and biocompatible and can circulate in the body for long periods of time [125]. Under the influence of US, the PFC experiences a phase transition from liquid to gaseous state. This transition generates a robust cavitation effect, which disrupts the external structure of the carrier, thereby facilitating drug release [126]. Cao et al. [104] encapsulated phase-changeable perfluoropentane (PFP) and DOX in lipid-based nanodroplets (LN) and PLGA-based nanodroplets (PN). Under low-intensity focused ultrasound (LIFU), PFP was vaporized into the gas phase, and the resulting nanodroplets were converted into microbubbles. The microbubbles rupture and release the loaded DOX. After two US treatments (1 MHz, 8 h: 5 W, 3 min; 1 MHz, 18 h: 8 W, 3 min), 80 % of the drug was released within 72 h, greater than the 50 % sustained release. In tumor-bearing mice, after two US-triggered treatments, the tumor volume remained stable for 12 days, indicating that US effectively facilitated drug release and significantly inhibited tumor growth.

Shen et al. [105] designed and prepared polyethylenimine-poly (N-isopropylacrylamide)-polyethylenimine (PEI_m_-PNIPAM-PEI_m_) triblock copolymers with an LCST that self-assembled into micelles at temperatures above the LCST (Fig. 7b). To form spherical nanogels, the PEI shells were crosslinked using crosslinking agents containing disulfide bonds, and these nanogels encapsulated DOX and US-sensitive perfluorohexane (PFH). Under US stimulation, PFH liquid transforms into microbubbles, resulting in the release of DOX as the bubbles burst. In addition, glutathione (GSH) present in the tumor specifically breaks down the S-S bonds, resulting in the uncrosslinking of the gel shell and the escape of DOX from the pores. In vitro drug release studies revealed that at 37 °C, less than 15 % of the total DOX was released within the initial 4 h. Following the introduction of GSH, approximately 40 % of DOX was released over the subsequent 4 h. Moreover, the application of US stimulation (25 kHz, 100 W/cm ^2^, pulse duration: 1.5 s) resulted in rapid and complete release of DOX.

Qin et al. [106] developed GSN-targeted phase-change PLGA nanoparticles (GSN-PLGA-PFH-DOX NPs) by encapsulating PFH and DOX within PLGA nanoparticles and functionalizing the surface with a gelsolin (GSN) monoclonal antibody. These nanoparticles are responsive to US, and upon exposure to LIFU, PFH undergoes a phase transition from liquid to gas, forming microbubbles. The oscillation and rupture of these microbubbles result in the release of DOX, which targets and eliminates cancer cells. During the LIFU-triggered phase transition, the size of the nanoparticles increased approximately 200-fold. In vitro drug release studies demonstrated that, without sonication, only about 29 % of the drug was released within the first 8 h. Following LIFU treatment (1 MHz, 8 W, 3 min), 60 % of the DOX was released in 8 h. Apoptosis assays indicated that the cell viability in the GSN-PLGA-PFH-DOX NP group was significantly reduced after LIFU irradiation, with more than 60 % of the cells undergoing apoptosis. Furthermore, the cytotoxicity of GSN-PLGA-PFH-DOX NPs was markedly higher than that of free DOX after LIFU treatment, suggesting that these nanoparticles are not only responsive to US, but also exhibit targeted delivery due to the presence of the GSN monoclonal antibody.

Li et al. [107] synthesized pH-responsive and US-responsive polydopamine (PDA) coated mesoporous silica nanoparticles (MSNs) (MSN@PDA). The particles were pH-responsive and US-responsive. The total DOX release from MSN@PDA was only 8.7 % within 48 h at pH of 7.4, 23.7 % at pH 5.5, and 71.2 % of the drug release at pH 3.0. This is because the solubility of DOX is inversely proportional to the pH of the solution, and thus acidic conditions are favorable for the diffusion of DOX from the pores into the medium. At the same time, under acidic conditions, PDA will be degraded, releasing the drug in the particles. Under the action of high-intensity focused ultrasound (HIFU), the cavitation effect leads to the generation, growth and rupture of many microbubbles in a very short time, and the energy generated at the instant of bubble rupture breaks the π-π non-covalent interactions or hydrogen bonding in the structure of the PDA, which accelerates the exchange of the substances around the carrier and promotes the release of DOX. After four HIFU stimulations (1.1 MHz, 100 W, 10 min), the pH 5.5 solution released 17 % of DOX within 4 h, and the pH 7.4 solution released 8 % of DOX within 4 h. These release percentages were much higher than those observed in the sustained release of DOX in both pHs, and a burst release of DOX was observed during sonication in both pHs. The pH and HIFU responsiveness of MSN@PDA particles for releasing DOX enable the on-demand release of the drug in the tumor environment.

Field et al. [108] developed US-responsive microcapsules using an aqueous two-phase system on a microfluidic chip. The outer phase of these microcapsules was composed of polyethylene glycol diacrylate, which prevented the passive release of the payload stored in the inner phase, dextran. Cavitation induced by focused ultrasound (FUS) disrupted the dextran inner phase, allowing for controlled drug release. The system exhibited effective US control, releasing approximately 0.552 ± 0.069 μg of fluorescent dextran after 16 FUS treatments (1.1 MHz, 150 w, 30 s) over 3 days, while untreated microcapsules released only 0.158 ± 0.013 μg.

Cullion et al. [109] co-coated TTX and fluorobutane into lipid microbubbles and induced the release of TTX under the action of high-frequency low-intensity ultrasound (HFLIU, 1 MHz, 0.1 W/cm ^2^, 5 min). The experimental results showed that rats had higher rates of neural blockade and motor blockade after the action of TTX + MB + HIFU compared to the injection of TTX solution, and the duration of both was longer (Fig. 7c). However, when bupivacaine was used as the guest molecule, US did not enhance the nerve block rate or motor block rate, nor did it prolong the duration of anesthesia. This lack of effect is likely due to bupivacaine’s hydrophobic nature, which affords it a greater ability to cross biological barriers, thereby reducing the impact of US in facilitating its delivery.

#### 2.2.3. Mechanism of US mechanical force-triggered drug release

In the presence of US, the rupture of microbubbles generates a mechanically elongated flow that drives the mechanical movement of the compound [127,128], and mechanochemically unstable bonds have low dissociation energies and are readily broken [129], which in turn disrupts the structural integrity of the carriers and releases drug molecules responsively. Simultaneously, the energy transmitted by US in the medium generates mechanical force, which acts on the drug carrier, leading to the destruction of its structure and subsequent drug release [113,114].

#### 2.2.4. US mechanical force-triggered drug release systems

Sun et al. [110] developed a US-responsive hydrogel featuring a double crosslinked nano-network structure. The backbone of the hydrogel was composed of four-armed polyethylene glycol acrylate (4 arm-PEG-Aclt) cross-linked with HA and this covalent cross-linking provided the hydrogel with substantial mechanical stability while significantly reducing drug release due to deformation. The phenylboronic acid coupled to HA formed a boronic acid ester bond with the anti-inflammatory compound tannic acid (TA), and the dynamic covalent boronic acid bond enabled the efficient release of TA triggered by US. US provides solvent-dynamic shearing of the bonds between TA and the polymer network, which is in principle capable of dissociating all borate bonds. When treated with US for 20 min per hour, about 8 % of TA was released in 10 h, whereas in a non-ultrasonicated gel, only 2 % of TA was released from the gel in 10 h. In addition, TA was usually released in an instantaneous burst after sonication, suggesting that the release of TA originated from dynamic fracture of the hydrogel network rather than permanent damage. In cellular experiments, macrophage inflammation was effectively suppressed after 10 US treatments, and in the absence of US-triggered TA release, the gel alone could not reduce macrophage inflammation.

Du and collaborators [111] engineered a DOX drug release system (BSA-ACVA-DOX NPs) utilizing US-controlled 4,4′-(diazene-1,2-diyl)bis (4-cyanopentanoic acid) (ACVA) cross-linked bovine serum albumin (BSA) nanoparticles. Upon US exposure, ACVA decomposes, leading to the degradation of BSA-ACVA-DOX NPs and the release of DOX for cancer treatment. The system demonstrated high US responsiveness, without US irradiation, only 5 % of DOX was released from the BSA-ACVA-DOX NPs within 10 min, with US (1 MHz, 2 W/cm ^2^), the nanoparticles released 16 % of DOX within the first 30 s, more than 56 % within 3 min, and 74 % after 10 min. The therapeutic efficacy of the BSA-ACVA-DOX NP was assessed, and MCF-7 cell viability decreased to 17 % after 3 min of sonication. In addition, turning on and off ultrasonography in vitro resulted in a programmable DOX release pattern within 2.0 cm of pork tissue. After the first minute of irradiation, around 30 % DOX was released, which could be halted by stopping US irradiation. Restarting the US for 2 min after 5 h resulted in 20 % DOX release. 10 % DOX release was seen again after 10 h of US irradiation, for a total of 70 % release on three triggers. This indicates that by controlling on and off the US, pulsed release of DOX can be achieved, allowing multiple intermittent chemotherapy treatments to be administered in a single dose (injection), depending on the therapeutic effect.

Darmawan and coworkers [112] evaluated a controlled drug delivery system using a magnetically managed self-rolling helical microrobot. It can navigate along the desired direction using a rotating magnetic field generated by an electromagnetic actuator, and upon reaching the location of the lesion, release a non-covalently bonded anticancer drug in a short period by US stimulation. During HIFU (1 MHz) treatment, the micro-robot is subjected to shear stresses generated by the HIFU, resulting in the breaking of hydrogen bonds of DOX, which is released from the micro-robot. After 60 s of HIFU stimulation, the drug release rate rapidly reached more than 90 % in pH 5.5 solution, while the cumulative drug release from the un-HIFU-stimulated microrobot was only 54.8 % in 24 h. Meanwhile, the significantly higher release rate of the microrobot in the acidic environment relative to the neutral environment suggests that the drug from the microrobot can be released more efficiently into the target tumor tissues with lower pH values, thus potentially reducing the side effects of the drug in normal tissues.

**Fig. 8.**
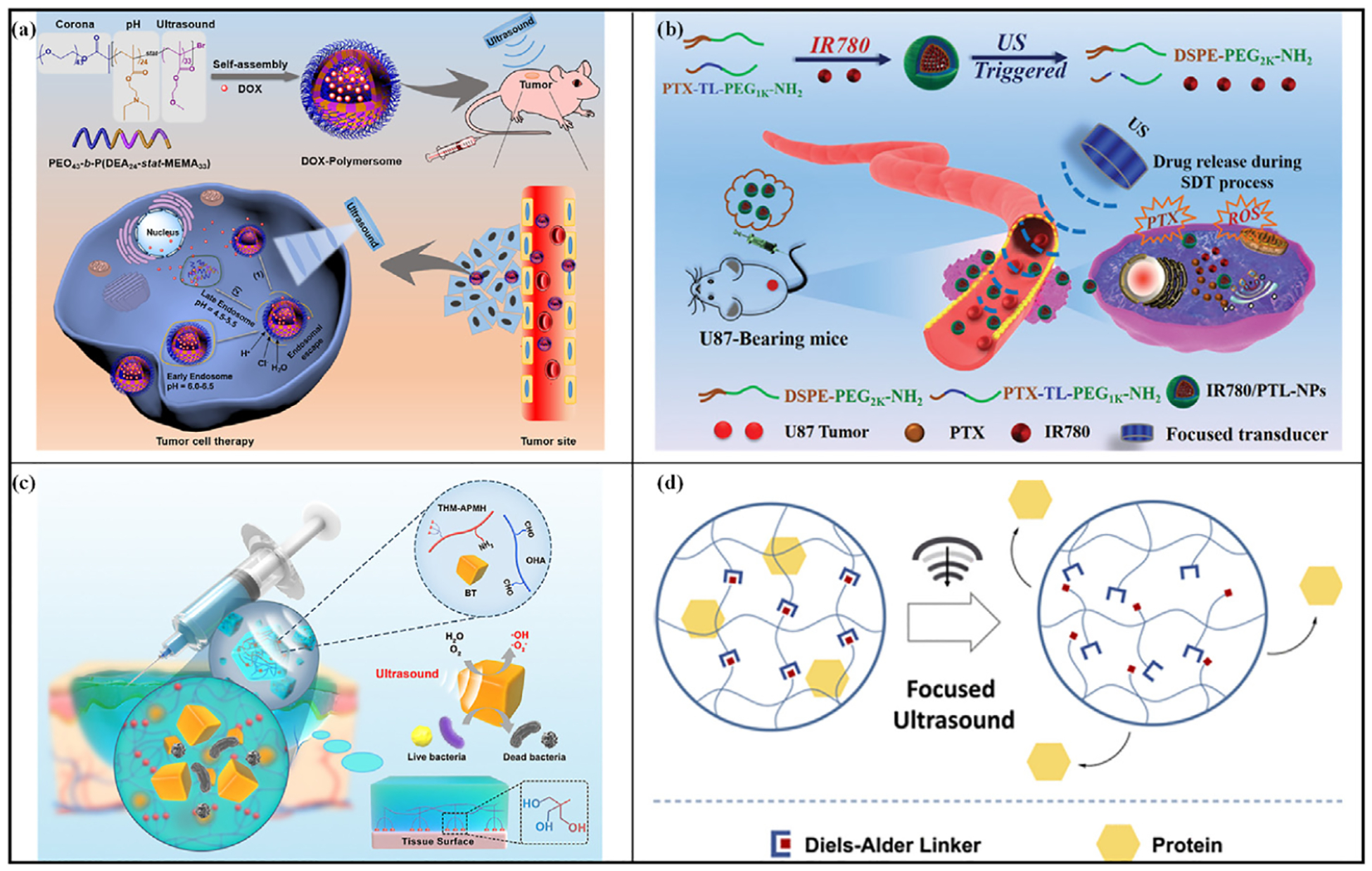
US mechanical force-triggered drug release: (a) Schematic illustration of US-responsive polymersomes for DOX delivery in tumor cells. Reprinted with permission from Ref. [113]. Copyright (2020) Elsevier; SDE triggered drug release: (b) Schematic illustration of the synergistic clearance of cancer cells by IR780/PTL-NPs combined with SDT-activated cascade chemotherapy. Reprinted with permission from Ref. [116]. Copyright (2019) John Wiley and Sons. (c) Schematic illustration of BT nanoparticles stimulated by US to generate ROS to kill bacteria. Reprinted with permission from Ref. [117]. Copyright (2023) Elsevier; US thermal effect-triggered drug release: (d) Schematic illustration of the gel undergoes an inverse Diels-Alder reaction under US to uncrosslink and release the protein. Reprinted with permission from Ref. [118]. Copyright (2022) American Chemical Society.

Wei et al. [113] created vesicles using a poly (ethylene oxide)-block-poly (2-(diethylamino)ethyl methacrylate)-stat-poly (methyl methacrylate) [PEO-b-P (DEA-stat-MEMA)] block copolymer and investigated the controlled release capabilities of this system both in vivo and in vitro, employing DOX as the model drug (Fig. 8a). Among them, PMEMA, as the US-responsive segment, can be treated by US to disrupt the vesicle structure by promoting chain movement, thus releasing the drug. The drug release test showed that at pH 7.4 and pH 6.0, the release of DOX was 48 % and 59 % after 24 h. The release rate increases immediately after applying US (20 kHz, 45 W, 3 min), and the release efficiency of DOX polymer vesicles increases from 48 % to 66 % at pH 7.4 and from 59 % to 73 % at pH 6.0 after 24 h, suggesting that both solution pH and US can induce the release of DOX from polymer vesicles. In vivo antitumor experiments in HeLa-loaded nude mice demonstrated that injection of drug-loaded polymer vesicles after US (1 MHz, 2.5 W/cm ^2^, 3 min) significantly reduced tumor growth in the mouse model, with a 95 % reduction in tumor volume within 12 days.

Sciurti et al. [114] explored the impact of US on square PLGA particles loaded with CUR. Their findings revealed that the mechanical force generated by US could disrupt the structure of these particles, resulting in a smaller diameter of CUR microplates (CURC-μPLs) thereby releasing CUR. Drug release indicated that the particles released about 10 % CUR within 30 min. Whereas the particles treated with 5 MHz, 200 kHz and 1 MHz US released about 15 %, 20 % and 25 % CUR, respectively and showing good sonication responsiveness.

#### 2.2.5. Mechanism of SDE-triggered drug release

Acoustic sensitizers can generate ROS, cavitation, bubbling, and thermotherapy effects in response to US, thereby modifying the structure of the carrier and initiating drug release [130]. In sonodynamic therapy (SDT), US waves activate acoustic sensitizers to generate ROS, effectively eliminating cancer cells [131]. The ROS produced through the SDE can disrupt ROS-sensitive structures or components within the carrier’s structure, facilitating the release of encapsulated drug molecules and enabling US-responsive drug release.

#### 2.2.6. SDE-triggered drug release systems

Bao et al. [115] devised a system for INS delivery mediated by US for diabetes treatment. In this setup, erythrocytes (ERs) encapsulating INS and PpIX were enclosed within a peptide hydrogel. Upon US exposure, PpIX generated ROS and interacted with phospholipid bilayer of ERs, causing the release of INS by opening the stomata of INS-PpIX@ER. Ceasing US irradiation closed the ER membrane’s stomata; thus, inhibiting INS release. With pulsed US (40 kHz, 0.3 W/cm ^2^, 10 s), the INS release exhibited a pulsatile pattern. In diabetic rats, hyperglycemia was managed by the INS-PpIX@ER hydrogel. A single injection of INS-PpIX@ER hydrogel decreased hyperglycemia in diabetic rats within 1 h. Following multiple US treatments (40 kHz, 0.3 W/cm ^2^, 30 s), blood glucose levels in diabetic rats remained within the normoglycemic range for up to 3 days.

Wu et al. [116] combined thioketal linkers (TL) with paclitaxel (PTX) and NH_2_-PEG1K-NH_2_ to produce PTX-TL-PEG1 k-NH_2_. This compound was then mixed with DSPE-PEG 2K-NH_2_ to fabricate nanoparticles loaded with the acoustic sensitizer IR780 (Fig. 8b). Upon exposure to FUS, IR780 generates a large amount of ROS, which triggers the disassembly of ROS-sensitive TLs, leading to the release of PTX from IR780/PTL-NPs. PTX synergistically kills cancer cells with ROS. In the U87 hormonal mouse model, controlled release of PTX under US (1 MHz, 0.4 W/cm ^2^, 3 min) irradiation significantly inhibited tumor growth, induced apoptosis, and led to a reduction of approximately 84.54 % in tumor volume over 17 days, demonstrating considerable therapeutic efficacy.

Liu et al. [117] developed a wound dressing with US responsiveness to enhance the healing of bacterial-infected wounds by embedding BaTiO_3_ (BT) into a hydrogel composed of N-[tris(hydroxymethyl) methyl]acrylamide, N-(3-aminopropyl)methacrylamide hydrochloride, and oxidized HA. When stimulated by US, BT generates a substantial amount of ROS, which effectively kills bacteria in the wound and promotes healing (Fig. 8c). US irradiation (1 MHz, 1.5 W/cm ^2^) of a rhodamine B (Rh.B) solution mixed with BT particles resulted in nearly complete degradation of Rh.B within 10 min, demonstrating the ability of BT particles to produce ROS under US irradiation. In vitro antimicrobial tests, the survival rate of *E. coli* decreased to 61.0 % after 2 min of US irradiation (1 MHz, 1.5 W/cm ^2^), and dropped to only 0.9 % after 10 min, indicating a clear time-dependent effect. Antimicrobial experiments on rat wounds revealed that, following US irradiation (1 MHz, 1.5 W/cm ^2^, 10 min), the group treated with a gel containing BT exhibited the most significant recovery, with a wound healing rate of 98.8 % by the ninth day.

#### 2.2.7. Mechanism of US thermal effect-triggered drug release

When US traverses a medium, a fraction of the acoustic energy is absorbed and subsequently converted into heat [132]. The amount of heat generated is influenced by the intensity and frequency of the ultrasound, as well as the absorption coefficient of the tissue [133]. Exposure of a temperature-sensitive carrier to ultrasound can lead to a rise in temperature, which may compromise the structural integrity of the carrier, cause the carrier to disintegrate or increase its permeability, thereby facilitating the release of the encapsulated drug.

#### 2.2.8. US thermal effect-triggered drug release systems

Arrizabalaga et al. [118] presented the cross-linking of chitosan with a Diels-Alder linker, yielding a US-responsive hydrogel (Fig. 8d). Upon application of focused US, the localized temperature increases within the gel triggered an inverse reaction in the Diels-Alder linker, leading to hydrogel disintegration and subsequent release of fluorescein-labeled bovine serum albumin (FITC-BSA). Experimental findings revealed a direct correlation between the protein release rate and the amplitude and duration of US. Evaluation of cytotoxicity indicated favorable biocompatibility of the gel.

Wu et al. [119] developed temperature-sensitive mPEG-PLGA-BOX hydrogels with mPEG, PLGA and 2,2′-bis(2-oxazoline) (BOX), and evaluated the release of small molecules (DOX) and macromolecules (FITC-dextran) from the hydrogels under the US. Under US (1 MHz, 0.4 W/cm ^2^, continuous), the gel mobility was enhanced with increasing temperature, and the release rates increased 70-fold for DOX and 83.9-fold for FITC-dextran compared with passive diffusion, indicating that US can trigger rapid release even for large molecules such as FITC-dextran. After stopping US, the release rate was the same as that during sustained release, suggesting that US-induced release is reversible, and that the hydrogel can be repeatedly triggered. The in vivo release of DOX in response to US was tested by subcutaneous injection into the back of rats, where the amount of DOX released from the hydrogel within 24 h after US (1 MHz, 0.4 W/cm ^2^, 3 min) treatment was 10 times the amount of DOX released from the sustained release. In addition, the residual amount of the hydrogel measured 7 days after subcutaneous injection was approximately 40 % of the injected volume, indicating good stability of the gel.

Wu described an US-responsive hydrogel based on NIPAM and N, N-methylenebisacrylamide (MBAA) that triggers the release of two macromolecules: BSA (66 kDa) and dextran (3–5 kDa) [120]. By adjusting the ratio of MBAA to NIPAM, the turbidity point of the hydrogel can be adjusted to 37 °C. In the presence of US, the temperature of the gel increases, when the temperature is higher than the turbidity point, the NIPAM chains become hydrophobic and aggregate, repelling water molecules bound to the NIPAM chains, leading to the separation of the polymer chains from the water and release of the encapsulated macromolecules. BSA release experiments have shown that when US or water bath heating raises the temperature of the hydrogel, the release of BSA increases, and the “flow effect” provided by the US (driving a steady flow in the fluid by absorbing acoustic energy) increases the release of BSA even further, resulting in a significantly higher release from US than from water bath heating. By adjusting the ratio of NIPAM and MBAA, the difference in BSA release between US (1 MHz, 3 W/cm ^2^, 3 min) and no US can be adjusted from 2.7-fold to 84-fold, and there is also a positive correlation between the intensity of sonication and release.

Levingstone et al. [121] developed a temperature-sensitive hydrogel with US-responsive properties by incorporating hydroxyapatite into an Alg hydrogel. This hydrogel is designed to release bone morphogenetic protein 2 (BMP-2) for bone repair. Sodium fluorescein and BSA were used as model compounds to investigate drug release kinetics. The study demonstrated that, while Alg hydrogels exhibited inherent US responsiveness, this property was significantly enhanced by the addition of hydroxyapatite. Moreover, the US responsiveness was found to be more pronounced for encapsulated macromolecules. During the release of BMP-2, subjecting the hydrogel to five US sessions (9.6 mW/cm ^2^, 25 % amplitude, 2.5 min per hour over 5 h) within 72 h resulted in a 10-fold increase in BMP-2 release compared to the release rate observed during sustained-release.

### 2.3. Magnetic field-triggered drug release

Magnetic fields have a wide range of applications in medicine, the magneto-thermal effect of iron oxide nanoparticles for the treatment of cancer has entered the clinical trial stage [134]. In recent years, large numbers of controlled drug-release systems using magnetic fields as external stimuli have been developed [135]. Magnetic fields as external stimuli have several advantages, including rapid response, remote controllability, precise control of drug release, no depth limitation, and noninvasive heat generation, while magnetic drug carriers can aggregate at the patient site under the action of magnetic fields to improve the targeting of the carriers [136–138]. Table 3 summarizes the mechanism of drug release triggered by a magnetic field. Based on the different triggering principles, magnetic field-triggered drug release systems can be categorized into two categories: magnetothermal effect-triggered drug release systems and magnetomechanical effect-triggered drug release systems.

#### 2.3.1. Mechanism of magnetothermal effect-triggered drug release

When MNPs are subjected to an alternating magnetic field (AMF), they generate heat through hysteresis loss in ferromagnetic or ferrimagnetic nanoparticles, and relaxation loss in superparamagnetic nanoparticles [153], this process results in a localized increase in temperature. In addition to its application in cancer therapy, this thermal effect can also be used in drug release, where the temperature increase will induce a phase change or hydrophobicity transition in thermo-sensitive drug carriers, thereby triggering the release of drugs [154].

#### 2.3.2. Magnetothermal effect-triggered drug release systems

**Fig. 9.**
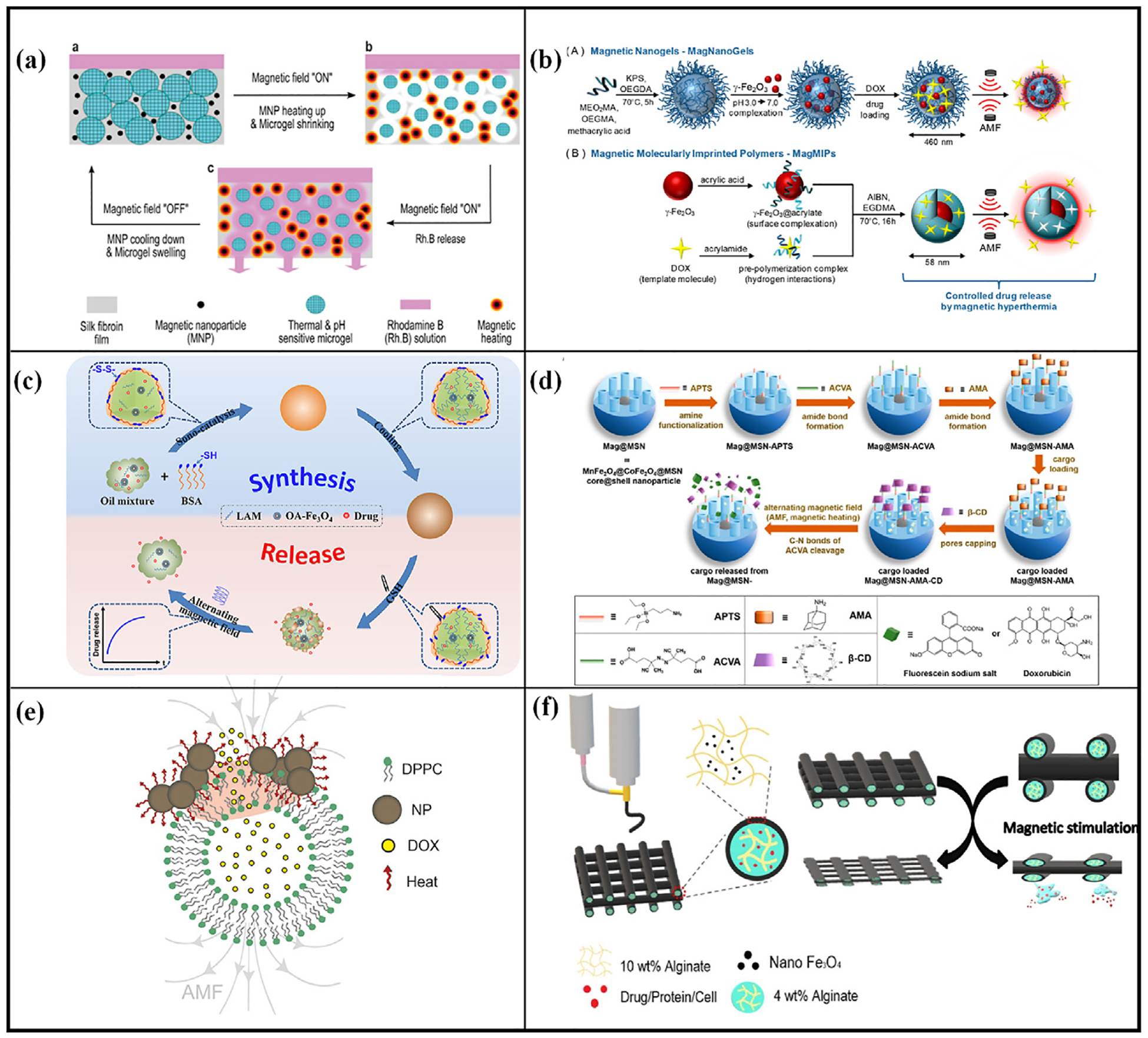
Magnetothermal effect-triggered drug release: (a) Silk composite membrane with MNPs and thermal/pH sensitive microgels for drug delivery controlled by an external magnetic field. Rh.B fluorescence dye is used as a model drug to demonstrate the working principle. Reprinted with permission from Ref. [139]. Copyright (1999–2024) John Wiley & Sons, Inc. (b) Schematic illustration of the preparation of MagNanoGels and MagMIPs and the triggering of DOX release under a magnetic field. Reprinted with permission from Ref. [141]. Copyright (2018) Multidisciplinary Digital Publishing Institute. (c) Schematic illustration for the sono-catalysis preparation and stimuli-triggered release of drug-loaded magnetic micro-organogel. Reprinted with permission from Ref. [142]. Copyright (2022) Elsevier. (d) Schematic illustration of the synthesis of azo snap-top core@shell MSNs and triggered release under an AMF. Reprinted with permission from Ref. [144]. Copyright (2019) American Chemical Society. (e) Schematic illustration of magnetic liposomes releasing drugs in AMF. Reprinted with permission from Ref. [146], Copyright (2020), American Chemical Society; Magnetomechanical effects-triggered drug release: (f) Schematic illustration of 3D printing of magnetically-driven drug delivery system and the magnetic-driven drug release. Reprinted with permission from Ref. [149], Copyright (2020), Elsevier.

Wang et al. [139] prepared a membrane with magnetic field adjustable permeability by doping PNIPAM microgel and MNPs into silk fibroin (Fig. 9a). When exposed to an AMF, the MNPs convert electromagnetic energy into thermal energy, causing a local temperature increase. This thermal effect induces shrinkage of the hydrogel, enhancing the permeability of the membrane and promoting the release of Rh.B. Under an AMF (111 kHz, 16 mT), Rh.B was released within 1 min. The release rate of Rh.B could be finely controlled (0.01–5.0 μg min ^−1^) by adjusting the microgel and MNP concentrations and the membrane thickness. Additionally, the release rate of Rh.B was higher under acidic conditions compared to neutral conditions, highlighting its potential therapeutic efficacy in the tumor environment.

Bi and colleagues [140] developed magnetic lipid microcapsules (MLM) containing lipid-coated MNPs. When subjected to an AMF, the superparamagnetic Fe_3_O_4_ nanoparticles within the capsules converted magnetic energy into thermal energy. This thermal effect altered the membrane’s permeability, facilitating the release of 5 (6)-Carboxyfluorescein (CF) from the MLM. With a Fe concentration of 20.0 μg/mL and an AMF intensity of 20 mT, triggered at a frequency of 5 kHz for 5 min, the maximum release rate of CF reached approximately 51.82 % after 120 min. In contrast, only about 4 % of CF was released in 120 min under natural conditions. Additional experiments showed that drug release was positively correlated with both the magnetic field frequency and the iron concentration.

Cazares-Cortes et al. [141] developed magnetic drug delivery systems comprising magnetic drug delivery gels (MagNanoGels) and nanoparticles (MagMIPs) for cancer therapy. The MagNanoGels are composed of iron oxide and thermosensitive polymers, while the MagMIPs feature core-shell nanoparticles, with iron oxide as the magnetic core and molecularly imprinted polymer as the shell (Fig. 9b). Under the influence of AMF, the localized temperature increase in MagNanoGels induces conformational changes in the polymer network, leading to drug release, MagMIPs release the drug by disrupting the hydrogen bonds between the drug and the polymer cage. In-vitro studies demonstrated that AMF-treated (335 kHz, 9 mT, 12.0 kA m ^−1^, 30 min) MagNanoGels and MagMIPs released 45 % and 60 % of DOX, respectively, after 4 h. In comparison, without AMF treatment, only approximately 24 % and 10 % of DOX were released from MagNanoGels and MagMIPs, respectively. Cytotoxicity assay showed a significant reduction in cell viability following AMF treatment (335 kHz, 9 mT, 12.0 kA m ^−1^, 30 min). Specifically, cell viability decreased from 54 % to 30 % for DOX-loaded MagNanoGels and from 88 % to 60 % for DOX-loaded MagMIPs, while free DOX maintained a survival rate of 92 % at the same dose. These results indicate that both nanomaterials are effective in promoting the internalization of DOX, with the therapeutic effect of using a magnetic field-triggered DOX release system being more pronounced than that of free DOX.

Li et al. [142] prepared a drug-loaded magnetic micro-organogel with a core-shell structure using oleic acid-modified Fe_3_O_4_ nanoparticles, N-lauroylalanine methyl ester (LAM), peanut oil and bovine serum albumin (BSA) (Fig. 9c). The gel has targeting properties, and GSH in the tumor microenvironment can reduce the disulfide bonds in BSA, destroying the structure of the gel and releasing the drug molecules in the core. The structure of the gel core is thermally reversible. Under an AMF, the gel temperature increases due to the magneto-thermal properties of Fe_3_O_4_. When the temperature exceeds the phase transition point, the gel collapses, breaking the hydrogen bonds between the gelling agents (LAM and oleic acid) and resulting in a substantial release of the drug. The experimental results showed that the microbial gel released coumarin 6 molecules rapidly in the presence of AMF and GSH together, releasing about 60 % in 40 h, while in the presence of GSH only, about 20 % of coumarin 6 molecules were released in 20 h.

Mirvakili et al. [143] prepared a polymeric magnetic nanocomposite microsphere made of MNPs and PLGA and demonstrated that these particles can be used for on-demand delivery with pulsatile release properties. In the presence of a magnetic field, MNPs can locally and rapidly increase the temperature. During excitation with a magnetic field (1.8 kW, 20 min), the release of sodium fluorescein was measured at 24 %, compared to a passive release of only 2.5–3% over the same period. After undergoing five magnetic field excitations over five days, a total of 61 % of the sodium fluorescein was released, demonstrating the microspheres’ effective responsiveness to the magnetic field.

Chen et al. [144] designed a stimuli-responsive nanoparticle platform. The platform consists of iron-doped oxide (MnFe_2_O_4_@CoFe_2_O_4_) nanoparticle cores and mesoporous silica shells, with temperature-sensitive ACVA acting as a linker to couple adamantane to the ionic surface, and adamantane-bound cyclodextrins acting as the gatekeepers (Fig. 9d). In the presence of an AMF, efficient magnetic heating from the superparamagnetic nanoparticle core cleaved the ACVA. Subsequently, the cyclodextrin gatekeeper disengaged, releasing the drug. Within 40 min at room temperature, only minimal fluorescein leakage (less than 5 %) was observed. However, the fluorescein release efficiency reached 10 % immediately after 1 min of triggering by the AMF (5 kW, 375 kHz). The release efficiency gradually increased with time until it reached a stable value after 70 min (total release efficiency of 23.6 %). In the presence of a pulsed magnetic field, two efficient triggers were observed, resulting in a final release of 80 % of the fluorescein. The results of DOX release were like those described above, with cumulative release rates of 35.6 %, 45.6 %, and 53.1 % of DOX after 2, 5, and 10 min of AMF excitation, respectively. Cell experiments also confirmed the stability of the carrier, and the cells could continue to grow without being triggered by the magnetic field, whereas when triggered by the magnetic field, the cells released DOX, producing significant cytotoxicity.

Hayashi et al. [145] fabricated tearable sponges (MDC sponges) composed of collagen, magnetite nanoparticles, and the anticancer drug DOX. The retardation of drug release was related to the degree of cross-linking of the sponge; a sponge cross-linked by heating for 6 h completely blocked drug release at 37 °C, and a sponge cross-linked by heating for 24 h completely blocked drug release at 45 °C. After AMF application, the sponge generates heat and raises the temperature from 37 °C to 45 °C within 15 min. The heat induces and regulates drug release from the sponge, with approximately 9 % of the DOX being released within 90 min after three triggers of the magnetic field, and the release of the drug slows down immediately after stopping the AMF. After three days of incubation with MDC sponges, the HeLa cells maintained 100 % viability. However, after 15 min of AMF treatment (74 Oe, 216 kHz), cell survival was 7.5 % ± 0.8 % and 2.1 % ± 0.3 % on day 3 and 5, respectively, indicating that the magnetic field was effective in stimulating DOX release.

Brollo et al. [146] prepared liposomes with iron oxide nanoparticles attached to the surface. In the presence of an AMF, the temperature of the iron oxide nanoparticles increased, the permeability of the membranes of liposomes was affected, and the loaded DOX was released (Fig. 9e). In vitro drug release showed that 20 % of DOX was released from liposomes after 1 h, while under the influence of an AMF (202 kHz, 30 m T, 1 h), the DOX released from magnetic liposomes increased by 27 %. Cell viability assays showed that cells co-cultured with magnetic liposomes survived above 80 %, but when triggered by a magnetic field (202 kHz, 30 m T, 1 h), cell survival was reduced to 17 % after 24 h, which was 70 % lower than in the absence of a magnetic field.

Xue et al. [147] developed a magnetically responsive drug release system by coloading superparamagnetic iron oxide nanoparticles (SPION) and DOX into alginate-chitosan microspheres. Comparing the effects of AMF and water bath as a heating source, the cumulative drug release after AMF (40 kA m ^−1^, 265 kHz, 10 min) exposure was twice as much as that of the water bath heating, a significant burst release was observed upon application of AMF, and when AMF was stopped, almost no DOX was released. In vitro cytotoxicity testing on MCF-7 breast cancer cells revealed that the thermal effects and drug release of the microsphere-based magnetic field resulted in 95.5 % cell death, approximately 1.5-fold and 1.1-fold greater than single magnetic heat or chemotherapy, respectively. An in vivo study in tumor-bearing nude mice showed that after AMF (265 kHz, 40 kA m ^−1^, 10 min) triggering, chemo-thermal synergistic treatment resulted in the disappearance of residual tumors within 12 days and no reappearance within the 40-day experimental period.

Fuller et al. [148] developed a magnetically controlled nanocarrier for temperature and magnetic field-controlled drug release by conjugating drug molecules to hexyl methacrylate-maleimide methacrylate via thermally unstable Diels-Alder bonds and combining them with SPION. The thermal effect of SPION, under the influence of a magnetic field, triggers the reverse reaction of the Diels-Alder bond, resulting in drug release. After 1 h of treatment with a pulsed magnetic field (346 kHz, 59.6 kA/m), the release of fluorescent molecules was 3.5 times higher than that of the sustained release, and after three triggers within 10 h, more than 6 % of the drug was released, demonstrating its ability to release multiple times.

#### 2.3.3. Mechanism of magnetomechanical effect-triggered drug release

Besides the magneto-thermal effect, MNPs also exhibit spatial repositioning in the magnetic field, resulting in a magneto-mechanical effect. This effect disrupts the cytoskeletal framework of cancer cells, leading to their demise [155]. Unlike the magneto-thermal effect, this process occurs within a magnetic field with very low frequency [156]. The mechanical and shear stresses generated during the movement of MNPs disrupt the carrier’s three-dimensional structure, increasing its porosity and free space, and enabling magnetic field-triggered drug release.

#### 2.3.4. Magnetomechanical effect-triggered drug release systems

Wang et al. [149] prepared hollow-fiber Alg/iron oxide nanoparticle scaffolds via coaxial 3D printing, encapsulating drugs, proteins, or living cells in the core portion (low concentration Alg gel). The guest molecule was extruded from the hollow fibers core by scaffold deformation induced by a magnetic field, resulting in magnetically driven on-demand release (Fig. 9f). Using DOX as a model drug, only 20 % of the drug was released in 144 h with magnetic field stimulation, but about 70 % of the drug was released in 144 h under undergoing 7 magnetic field stimulations (0.42 T, 1 min). The release of BSA was similarly greatly boosted by the magnetic field, and the protein was fully released after eight magnetic field stimulations over 144 h. Furthermore, since the whole scaffold construction and magnetic stimulation process occurs under benign circumstances (room/body temperature, no organic solvents), living cells can be directly encapsulated in the core gel, allowing for on-demand release for cell therapy and tissue engineering. In the absence of stimulation, the substantial hollow fiber wall slows cell release from the core gel. Implantation of the scaffold subcutaneously in mice under magnetic field (0.42 T, 1 min) stimulation effectively triggered the release of DOX twice within 48 h and showed a significant increase in DOX fluorescence after magnetic stimulation, indicating burst release.

Nguyen and colleagues [150] synthesized magnetite Fe_3_O_4_ nanoparticles by co-precipitation and loaded them into sodium alginate microbeads containing berberine. Interestingly, the magnetic field was effective in preventing drug release. Because of their superparamagnetic properties, the MNPs in the sodium alginate microbeads were in a state of zero magnetization in the absence of static magnetic field, resulting in a typical diffusion pattern in the drug release profile. When a static magnetic field was applied, the magnetic moments of the nanoparticles aligned with the magnetic field, resulting in immediate aggregation of the MNPs and rapid reduction in the porosity of the beads. As a result, the drug molecules were restricted to the Alg bead network, which significantly reduced the drugs diffusion into the release medium. In the absence of magnetic field, the carriers released about 80 % of the drug in acetate buffer solution and 55 % in phosphate buffer solution within 25 min, whereas the rate of drug release decreased significantly after the application of a static magnetic field (3.5 T, 45 min), and the rate of drug release was rapidly increased after the cessation of the magnetic stimulation, which demonstrated the superior controllability.

Hu and co-workers [151] prepared pH/magnetic field-driven hydrogels using salicin-g-poly (vinyl acetic acid-acrylic acid 2-hydroxyethyl ester) (PVH) copolymers with Fe_3_O_4_@Agarose nanoparticles. The hydrogels have pH/magnetic field-triggered release properties that could be accelerated by weakly acidic conditions or an external magnetic field. Under acidic conditions (pH 4.5), PVH and DOX were protonated, and there was a weak electrostatic attraction between the drug and the hydrogel carrier, and DOX was easily released through the hydrogel matrix. The experimental results showed that the cumulative release of DOX could reach 76 % after 64 h at pH 4.5, which was much higher than the cumulative release at pH 6.8 and 7.4 (16.9 % and 10.5 %, respectively). The MNPs tended to be ordered in response to the applied magnetic field, resulting in the movement of Fe_3_O_4_@Agarose nanoparticles, widening the gel network, and promoting DOX release. In the presence of an applied magnetic field (2200 G), 91.3 % of DOX was released within 60 h, which was 1.2 times higher than that in the no magnetic field condition. Cytotoxicity studies showed that the released DOX had excellent killing effect on A549 cells, with the highest growth inhibition (up to 76.4 %).

Kondaveeti et al. [152]. developed a magnetically sensitive hydrogel from calcium ion-crosslinked Alg and xanthan gum (XG) modified by MNP. The suatained drug release and on-demand release of the gel under static external magnetic field stimulation was investigated using the anti-Parkinson’s disease drug levodopa (LD) as a model drug. The amount of LD released from Alg-XG/MNP/LD was 45 ± 5 % after 30 h under dark conditions at pH 7.4 and 8 °C, whereas the amount of LD released from Alg-XG/MNP/LD in the presence of an EMF (0.4 T) was (64 ± 6)%, which was attributed to the fact that the magnetic field facilitated the orientation of the MNP, caused local mechanical vibration of the hydrogel, and stimulated the hydrogel to release of LD. Considering that LD is the most effective drug for the treatment of motor symptoms in Parkinson’s disease, which is associated with the loss of dopamine-producing brain cells and the subsequent lack of this neurotransmitter in the synaptic gap, the effect of an external magnetic field-triggered LD release on the neuronal cells was evaluated. The results showed a 51 % increase in human neurons when cultured on Alg-XG/MNP + LD scaffolds in three days in the presence of an electromagnetic field.

### 2.4. Temperature-triggered drug release

Temperature is one of the most frequently utilized stimuli, and thermotherapy has been employed to augment drug permeability in tissues and enhance tumor sensitivity to chemotherapeutic agents [157, 158]. Employing temperature as an exogenous stimulus to initiate drug release offers the advantages of straightforward control and ease of operation [159]. Additionally, the robust temperature tolerance of human skin allows for the direct application of a cold or heat source to adjust the ambient temperature of subcutaneous thermoreceptor carriers, thereby inducing drug release [160,161]. Table 4 provides a summary of temperature-sensitive drug release systems.

#### 2.4.1. Mechanism of temperature-triggered drug release

Thermosensitive materials are capable of altering their physical or chemical properties in response to temperature fluctuations [169]. When these materials are employed as drug carriers, external regulation of the carrier’s temperature can induce various microscopic changes, such as transitions between hydrophilic and hydrophobic states, disruption of thermosensitive bonds, and increased molecular motion [170]. These microscopic alterations can, in turn, provoke macroscopic effects including phase transitions, changes in membrane permeability, and disintegration of the carrier, facilitating and triggering the release of the encapsulated drug [171].

#### 2.4.2. Temperature-triggered drug release systems

**Fig. 10.**
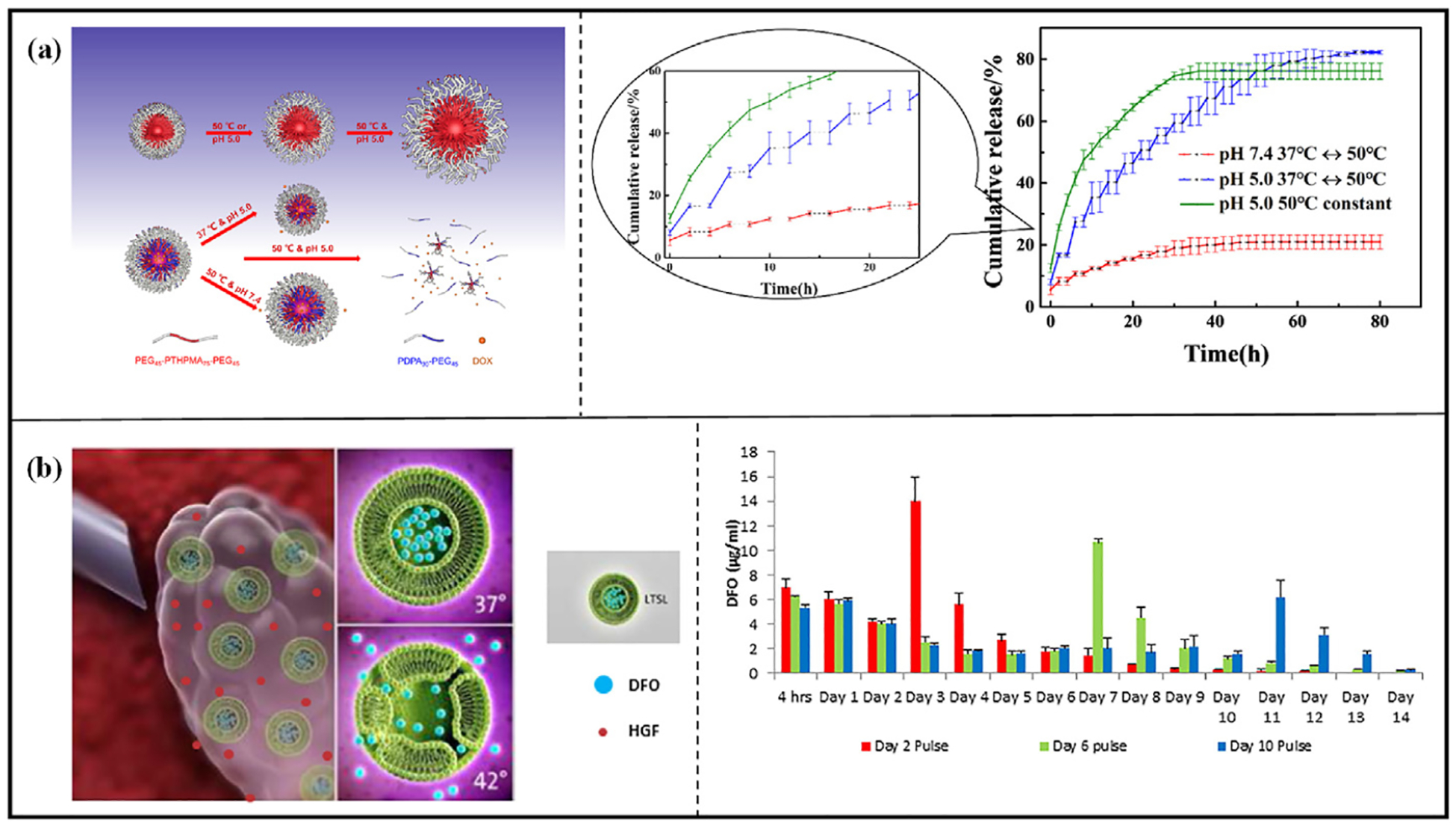
Temperature-triggered drug release: (a) Left: Schematic illustration of shape changes of micelles under pH and temperature stimulation. Right: At pH 5.0, the release profile of DOX from micelles was stepped by temperature-controlled cycling (37 °C/2 h, 45 °C/2 h). Reprinted with permission from Ref. [162]. Copyright (2021) American Chemical Society. (b) Left: Schematic illustration of DFO release from at 42 °C. Right: High-temperature pulses (42 °C, 1 h) on days 2, 6, or 10 are effective in triggering DFO release up to three days after stimulation. Reprinted with permission from Ref. [168]. Copyright (2016) Elsevier.

Lei et al. [162] developed a pH/temperature bi-responsive hybrid micelle system using a combination of poly (ethylene glycol)-poly (tetrahydropyranosyl methacrylate)-poly (ethylene glycol) (PEG-PTHPMA--PEG) triblock copolymer and poly (diisopropylaminoethylene glycol) methacrylate-poly (ethylene glycol) (PDPA-PEG), creating a dual-lock drug delivery system (Fig. 10a: Left). In this system, PTHPMA responds to temperature changes by undergoing hydrolysis of the THP group at elevated temperatures, which converts the PTHPMA from hydrophobic to hydrophilic. PDPA responds to pH changes; under acidic conditions, PDPA becomes protonated, causing the micelle core to loosen. The combined effects of these two components lead to the disintegration of the micelles and subsequent release of DOX. In vitro drug release studies demonstrated that the dual-locking mechanism achieved optimal release of DOX at 45 °C and pH 3.0. The release profile exhibited a stepwise pattern when cycling through temperature controls at pH 5.0 (37 °C/2 h, 45 °C/2 h) (Fig. 10a: Right). Cytotoxicity testing revealed that the DOX-loaded micelles exhibited the highest cytotoxicity at 45 °C, with HeLa cell survival decreasing by 23–72 % after 1 h.

Kang et al. [163] engineered a tumor-targeting system by grafting a copolymer of NIPAM and allylamine onto the surface of FePt clusters, with FA attached for tumor targeting, resulting in PNIPAM-FePt nanoclusters. The drug release mechanism is based on the reversible phase transition of PNIPAM, which changes from a hydrophilic to a hydrophobic state as the temperature increases. This temperature-induced transition causes the polymer to shrink, thereby expelling the loaded molecules from the nanoclusters and facilitating drug release. Experimental results indicate that the phase transition temperature of the nanoparticles can be tuned by adjusting the proportion of copolymer grafted onto the surface. The nanoparticles exhibit heightened temperature sensitivity for amphiphilic drugs within the temperature range of 25–37 °C. Fluorescence intensity measurements of nanoclusters loaded with amphiphilic Rh.B showed a decrease with increasing temperature, indicating effective expulsion of Rh.B molecules due to polymer shrinkage. Conversely, the release of hydrophobic Nile red molecules was minimally affected by temperature changes. This is because hydrophobic molecules tend to aggregate within the inner part of the clusters, and while elevated temperature causes the outer molecules to be expelled, the remaining molecules are reabsorbed and encapsulated by the polymer due to their hydrophobic nature. Additionally, temperature cycling experiments demonstrated the reversible nature of the temperature-responsive behavior of the nanoclusters.

Li et al. [164] developed temperature-sensitive liposomes (c-LIP-WSG) targeting ovarian cancer by coupling phospholipid molecules with an ovarian cancer-targeted peptide ligand, WSG, and integrating organic-inorganic hybrid cerasome-forming lipid using a thin-film hydration method. In vitro release studies revealed that at 37 °C, c-LIP-WSG released only 16.5 % of DOX within 90 min. However, at 42 °C, the release increased to over 80 %, indicating that c-LIP-WSG exhibits temperature-sensitive release properties. Additionally, in SKOV-3 hormone-sensitive nude mice, c-LIP-WSG demonstrated a strong targeting effect, preferentially accumulating in the tumor region.

Wang et al. [165] devised a novel formulation of cold-responsive nanoparticles (HCPN-CG) comprising HA, chitosan, poly (N-isopropylacrylamide-co-butyl acrylate (PNIPAM-B), and Pluronic F127 for the targeted co-delivery of chemotherapeutic agents and photothermal agents (ICGs) for breast tumor treatment. PNIPAM-B, being temperature-sensitive, dissolves at low temperatures, leading to rapid decomposition of the nanoparticles at temperatures below 12 °C. After 5 min of cooling on ice, more than 80 % of CPT was released from the nanoparticles. However, only approximately 2 % of the CPT was released within 5 h at 37 °C, indicating excellent temperature responsiveness. ICG acts as a photothermal agent under NIR light irradiation, and the thermal effect assists CPT in killing cancer cells. In vivo experiments in mice with MDA-MB-231 human mammary tumors showed that after injection of HCPN-CG nanoparticles into the tail vein, the HCPN-CG nanoparticles would accumulate in the tumor region with good targeting properties. The tumor growth was significantly inhibited within 30 days by stimulation with low-temperature ice (5 min) and NIR laser irradiation (1 W/cm ^2^, 2 min), and no side effects were observed.

Xu et al. [166] and others created a sandwich membrane using temperature-responsive micelles and HA biopolymers based on hydrogen-bonded layer-by-layer self-assembly, and introduced temperature sensitivity to the composite membrane by depositing block copolymer micelles with PNIPAM cores into the multilayered membrane for the treatment of third-generation non-small-cell lung cancer, using osimertinib as a model drug molecule to study the controlled release of the drug. As temperature changes, PNIPAM undergoes a hydrophilic-hydrophobic transition and the interaction with the guest molecule changes, resulting in the responsive release of the guest molecule. The release half-life of osimertinib at 25, 37 and 55 °C with a solution pH of 2.2 was 2, 7 and 10 h, respectively, with higher release rates at lower temperatures. When the membrane was placed at 55 °C, approximately 80 % of the osimertinib was released after three temperature stimuli (25 °C, 1 h) over 15 h, with a maximum single release of 28 %.

Chen et al. [167] used chitosan particles as the base material, in situ gel generation (PNINAM) was performed to encapsulate fibroblast growth factor (FGF) in the particle gaps to develop biomass composite anti-protein particles for wound healing. In vitro experiments with FITC-BSA as a model molecule showed that the FITC-BSA was released upon temperature stimulation (45 °C, 5 min), with a maximum single release of approximately 60 % of the FITC-BSA. In vivo experiments demonstrated that rats treated with the FGF-containing particles at the wound site exhibited the greatest amount of granulation tissue, the highest collagen deposition, and the most extensive vascular structures within seven days.

O’Neill et al. [168] developed a temperature-sensitive gel drug carrier (Lipogel) for the promotion of vascular endothelial growth factor production by co-mixing lysophospholipid-based thermosensitive liposomes (LTSL) containing desferrioxamine (DFO) with chitosan-based gels (Fig. 10b: Left). In vitro drug release demonstrated that encapsulation of DFO by LTSL effectively slowed down the sustained-release rate, with only about 50 % of DFO being released from the gel at 37 °C over 72 h, compared to over 90 % for unencapsulated DFO. The temperature sensitivity of Lipogel was evaluated and high-temperature pulses (42 °C, 1h) on days 2, 6, or 10 were effective in triggering the release of DFO for up to three days post-stimulation (Fig. 10b: Right). In addition, the amount of DFO released depended on the duration of the pulse, with a gradual increase in the amount of DFO released in a single pulse over 24 h as the pulse duration was extended from 1 min to 30 min, and saturation of DFO release in a single pulse was reached after more than 30 min.

## 3. Conclusions and future directions

Externally triggered drug delivery systems provide unique advantages in the on-demand release of drugs for the treatment of diabetes, cancer, and chronic pain etc. This review summarizes recent advances in stimulus-responsive drug delivery systems, including light, US, magnetic field, and temperature-based drug delivery. Table 5 summarizes the advantages and limitations of each system. Each delivery system is evaluated based on its stimulus responsiveness and controlled release repeatability. In addition, the rationale and design concepts for each system’s on-demand drug delivery are emphasized.

Despite considerable progress, various challenges persist, preventing the externally triggered drug delivery systems from being clinically considered. Firstly, drug delivery systems experience passive drug release. Many of the stimuli-responsive drug delivery systems rely on the same mechanism to encapsulate drugs as regular sustained-release systems, which utilize physical interactions (van der Waals forces, hydrogen bonding, Coulombic forces) with the drug molecules to maintain drug encapsulated. These physical interactions are typically weak. Consequently, there is a tendency for passive drug release in the absence of an external stimulus. Similarly, hydrophilic drugs, despite initial effective encapsulation in liposomes and polymersomes, tend to undergo passive release over time due to their inherent hydrophilicity. This passive release, post-injection, leads to an immediate but undesired drug effect. Moreover, this basal drug release reduces the drug available for subsequent trigger events. Binding drug molecules to the carrier through external stimuli-sensitive chemical bonds represents a promising strategy for mitigating passive drug release.

Secondly, the reproducibility of the stimulus response poses another challenge in the current development of the field. Limited reproducibility manifests in several ways: triggering events can only be repeated a finite number of times, and they are constrained to occur within a short timeframe post-initial injection. These constraints diminish the advantages of responsive release compared to traditional needle injections. The lack of reproducibility can be attributed to various factors, including inadequate local retention time of the drug carrier, the depletion of drug dose within the carrier after initial passive release, and residual drug remaining within the carrier even after multiple treatments. Addressing these limitations should be a priority in the future design of externally triggered drug delivery systems to enhance the reproducibility of stimulus response.

Thirdly, intrinsic systemic/local toxicity and the long-term accumulation of drug delivery systems in the human body represent additional limiting factors. The development of stimuli-responsive drug delivery systems with high biocompatibility and degradability is crucial for their clinical medical applications.

## Figures and Tables

**Table 1:** Summary of light-triggered drug release systems.

Style	Irradiation parameter	Mechanisms of light-triggered drug release	Reference
PDE-triggered drug release	808 nm, 2 W/cm^2^, 15 min	ROS breaks the diselenide bond	[34]
	660 nm, 300 mW/cm^2^, 5 min	ROS breaks the diselenide bond	[35]
	633 nm, 50 mW/cm^2^, 10 min	ROS breaks the TK bonds	[36]
	808 nm, 0.5 W/cm^2^, 10 min	ROS breaks the TK bonds	[37]
	660 nm, 200 mW/cm^2^, 5 min	ROS breaks the TK bonds	[38]
	650 nm, 0.5 W/cm^2^, 10 min	ROS alters the hydrophobicity of liposome membranes.	[39]
	X ray, 4 Gy, 10 min	The nanoparticle shell layer changes from amphiphilic to hydrophilic	[40]
	730 nm, 75 mW/cm^2^, 5 min	ROS peroxidizes liposomes and alters membrane permeability	[41]
	730 nm, 50 mW/cm^2^, 15 min	ROS peroxidizes liposomes and alters membrane permeability	[42]
PTE-triggered drug release	808 nm, 0.5 W/cm^2^, 10 min	ICG converts light energy into heat, causing a phase change in the drug-carrying crystal	[43]
	808 nm, 1.5–2.0 W/cm^2^, 7 min	ICG converts light energy into heat, causing a phase change in fatty acids	[44]
	785 nm, 1 W/cm^2^, 10 min	Nanocarriers undergo a phase transition that triggers the release of CPT-11	[45]
	808 nm, 0.5 W/cm^2^, 10 min	The PTE of PDA disrupts the intermolecular forces between Cip and PDA	[46]
	808 nm, 2 W/cm^2^, 2 min	High temperatures lead to degradation of micelles	[47]
	808 nm, 650 mW/cm^2^, 7 min	Elevated temperatures cause phase transitions in the crosslinked structures causing the gel to disintegrate	[48]
	808 nm, 5 min	Phase transition in temperature-sensitive polymer triggers drug release	[49]
	808 nm, 272 mW/cm^2^, 10 min	The PTE of GNR causes a phase transition in liposomes to trigger drug release	[50]
	808 nm, 570 mW/cm^2^, 30 min	The PTE of AuNS causes the copolymer nanogel particles in the membrane layer to shrink, increasing membrane permeability	[51]
	730 nm, 200 mW/cm^2^, 3 min	The PTE of GNR causes a phase transition in liposomes to trigger drug release	[52]
	Blue light, 474 mW/cm^2^, 1 h	Phase transition in temperature-sensitive gel beads trigger drug release	[53]
	808 nm, 1.0 W/cm^2^, 5 min	BP converts light energy into heat, softening and melting hydrogel nanostructures	[54]
	808 nm, 1 W/cm^2^, 10 min	nGO nanoparticles transform light energy into heat energy and induce phase transition in thermosensitive liposomes	[55]
Photo-cleavage triggered drug release	650 nm, 0.2 W/cm^2^, 30 min	Photodissociation of Cy leads to shell layer deshielding of MSNs	[56]
	532 nm, 1.12W/cm^2^, 5 min	The linker S, S-tetrazine cleaves under light, causing the hydrogel to degrade	[57]
	400 nm, 300 mW/cm^2^, 2 min	The carbamate bond between the tetracaine and the carrier is broken, which releases the drug molecule	[58]
	400 nm, 50 mW/cm^2^, 30 s	The coumarin caging group was cleaved after light exposure and the targeting activity of the peptide was restored.	[59]
	400 nm, 300 mW/cm^2^, 2 min	The carbamate bond between the naloxone molecule and the carrier is broken, which releases the drug molecule	[60]
	505 nm, 50 mw/cm^2^, 5 min	The carbamate bond is broken, and the carrier decomposes to release the drug	[61]
	254 nm, 4.5 mW/cm^2^, 5 min	The coumarin-based linker undergoes a retro [2 + 2] cycloaddition reaction with 5-FU to release 5-FU	[62]
	365 nm, 1 mW/cm^2^, 5 min	O-nitrophenyl methyl ester in the linker decomposes into carboxylic acid and O-nitrobenzaldehyde	[63]
UCNPs triggered drug release	980 nm, 0.75 W/cm^2^, 10 min	UCNPs convert NIR light into UV light, causing continual rotational-reversal motion of the micelles	[64]
	980 nm, 0.30 W/cm^2^, 5–20 min	UCNPs convert NIR to UV light, break thioether linkers, and trigger drug release	[65]
	980 nm, 1.5 W/cm^2^, 1 min	UCNP converts NIR into UV light, which cleaves the S-NO bond in nitrosothiols, releasing NO	[66]

**Table 2:** Summary of US-triggered drug release systems.

Style	Irradiation parameter	Mechanisms of US-triggered drug release	Reference
Cavitation effect- triggered drug release	1 MHz, 5 W, 3 min; 1 MHz 8 W,3 min	Microbubbles disrupt the integrity of the liposome, releasing loaded DOX	[104]
	25 kHz, 100 W/cm^2^, 1.5 s	Microbubbles disrupt the integrity of the gel, releasing loaded DOX	[105]
	1 MHz, 8W, 3 min	Microbubbles disrupt the integrity of the nanoparticles, releasing loaded DOX	[106]
	1.1 MHz, 100 W, 10 min	The energy generated by the bursting of the microbubbles breaks the non-covalent π-π interactions or hydrogen bonds between DOX and the PDA, releasing DOX	[107]
	1.1 MHz, 150 W, 30 s	Cavitation-induced breakdown of the dextran phase and part of the PEGDA phase	[108]
	1 MHz, 0.1 W/cm^2^, 5min	Microbubbles disrupt the integrity of the liposome, releasing loaded TTX	[109]
Mechanical force-triggered drug release	/	US disrupts dynamic borate bonds between TA and hydrogels	[110]
	1 MHz, 2 W/cm^2^, 3 min	US promotes the decomposition of crosslinks, leading to nanoparticle degradation	[111]
	1 MHz, 20 Vpp, 60 s	HIFU generates shear stresses that lead to hydrogen bond breakage	[112]
	1 MHz, 2.5 W/cm^2^, 3 min	US promotes the movement of responsive polymer chain segments to disrupt vesicle structure	[113]
	1 MHz, 5 MHz, 200 kHz, 30 min	US destroys the external structure of polymer particles	[114]
SDE-triggered drug release	40 kHz, 0.3 W/cm^2^, 30 s	ROS interacts with the phospholipid bilayer of erythrocytes to generate stomata	[115]
	1 MHz, 0.4 W/cm^2^, 3 min	ROS breaks down TL and release PTX	[116]
	1 MHz, 1.5 W/cm^2^, 10 min	US triggers the production of ROS, which act as active molecules to eliminate bacteria	[117]
Thermal effect- triggered drug release	1.5 MHz, peak positive pressures: 75 MPa, 5 min	Reverse Diels-Alder reaction occurs at elevated temperatures, hydrogel breaks down	[118]
	1 MHz, 0.4 W/cm^2^, 3 min	US induces a localized temperature increase causing the gel to undergo a phase change	[119]
	1 MHz, 3 W/cm^2^, 3 min	At elevated temperatures, the temperature-sensitive polymer chains undergo a hydrophobic transition, shrinking the gel volume and extruding the drug molecules	[120]
	9.6 mW/cm^2^, 25 % amplitude, 2.5 min	Thermal effects induce changes in the hydrophobicity of temperature-sensitive polymers, resulting in a reduction in the compactness of the gel	[121]

**Table 3:** Summary of magnetic field-triggered drug release systems.

Style	Irradiation parameter	Mechanism of magnetic field-triggered drug release	Reference
Magnetothermal effect-triggered drug release	111 kHz, 16 mT, 1min	Increased local temperature causes microgel contraction to release the drug	[139]
	5 kHz, 20 mT, 5 min	Elevated temperature alters the permeability of liposome membranes	[140]
	335 kHz, 9 mT, 12.0 kA m^−1^, 30 min	Elevated temperature changes the conformation of the polymer in the gel and breaks the hydrogen bonds between DOX and nanoparticles	[141]
	AMF	Increased temperature breaks the hydrogen bonds between the gelling agents, causing the gel to undergo a phase change	[142]
	1.8 kW, 20 min	Increased temperature increases polymer chains mobility, which expands free space inside the polymer network	[143]
	375 KHz, 5 kW, 1 min	Localized thermal effects cause the ACVA to decompose and the shell gatekeeper to leave	[144]
	216 kHz, 74 Oe, 15 min	Thermal effect disrupts the crosslinked structure of the sponge	[145]
	202 kHz, 30 mT, 1 h	Elevated temperature increases the permeability of liposome membranes	[146]
	265 kHz, 40 kA m^−1^, 10 min	Localized temperature increases cause phase change in the gel	[147]
	346 kHz, 59.6 kA/m, 1 h	Elevated temperature breaks the Diels-Alder bond	[148]
Magneto-mechanical effect-triggered drug release	0.42 T, 1 min	Under magnetic field, the guest molecules are extruded from the core of the hollow fibers by the deformation of the stent	[149]
	3.5 T, 45 min	Aggregation of magnetic nanoparticles in a magnetic field rapidly reduces the porosity of sodium Alg beads to prevent drug release	[150]
	2200 G	Orderly arrangement of magnetic nanoparticles increases the free space inside the gel and facilitates DOX release	[151]
	0.4T	Orderly arrangement of magnetic nanoparticles increases the free space inside the gel and facilitates LD release	[152]

**Table 4:** Summary of temperature-triggered drug release systems.

Irradiation parameter	Mechanism of temperature-triggered drug release	Reference
45 °C, pH 5.0	Elevated temperature led to changes in the hydrophobicity of the micelles and consequent disintegration	[162]
25 °C, 37 °C	As the temperature rises, the polymer shrinks, causing the loaded molecules to extrude from the nanoclusters	[163]
42 °C, 90 min	Liposomes undergo a phase transition above the critical transition temperature	[164]
0 °C, 5 min	Weakened polymer hydrophobicity leads to nanoparticle disintegration.	[165]
25 °C, pH = 2.2, 1 h	Lower temperature increases the hydrophilicity of the polymer and weakens the interaction with the guest molecule	[166]
45 °C, 5 min	Temperature-sensitive polymers shrink on warming, extruding the encapsulated drug	[167]
42 °C, 60 h	Increased permeability of temperature-sensitive liposome membranes at high temperatures	[168]

**Table 5:** Advantages and disadvantages of externally triggered drug release systems.

Categories	Advantages	Disadvantages	Reference
Light-triggered drug release	Low cost, exquisite temporal, and spatial control. UV: High energy, wide range of activatable moieties. NIR: Tissue penetration can be up to 10 cm deep. Good biosafety.	UV: Poor tissue penetration only penetrates superficial tissues at the micron level. High phototoxicity, prone to tissue damage and skin aging. NIR: Low energy, not enough to cause a photo-responsive chemical reaction. Lower efficiency and prolonged exposure may cause damage to surrounding healthy tissue due to unnecessary overheating.	[10,29,172–176]
US-triggered drug release	High spatial discrimination, and strong tissue penetration ability, frequencies below 1 MHz can penetrate most of bodies. Can promote drug absorption and improve drug efficacy. Low cost.	Cavitation and thermal effects may damage tissue. Difficult to apply uniformly over large areas. Difficult to target moving organs. Skeletal strongly attenuated US.	[176–179]
Magnetic field-triggered drug release	Can be controlled remotely. Ability to precisely control the location and rate of drug delivery. Strong tissue penetration with no penetration depth limitation.	Magneto-thermal effects may irreversibly damage tissue. Accumulation of MNPs may block blood vessels and cause tissue toxicity. High cost and complex equipment. Not suitable for patients with magnetically sensitive materials (pacemakers) in their bodies.	[176–182]
Temperature-triggered drug release	Low requirements for the trigger device, easy to operate Low economic cost, simple manufacturing, and formulation.	Limited depth of penetration. Cold or heat sources need to be close to the skin and may damage tissue.	[160,161,176]

## Abbreviations

Alg: Alginate
AMF: Alternating magnetic field
BT: BaTiO_3_
BP: Black phosphorus
BOX: 2,2′-bis(2-oxazoline)
BMP-2: bone morphogenetic protein 2
BSA: bovine serum albumin
BUPI-HCl: Bupivacaine hydrochloride
CPT: Camptothecin
CPT-11: Camptothecin-11
CF: 5(6)-Carboxyfluorescein
CPPs: Cell-penetrating peptides
Cip: Ciprofloxacin
IMTD: Co-assembly of indocyanine green and mannose-thioketal-doxorubicin conjugate
CUR: Curcumin
CURC-μPLs: Curcumin-microplates
β-CD: β-Cyclodextrin
DFO: Desferroxamine
DEX: Dexamethasone
DMED: Dexmedetomidine
ACVA: 4,4′-(diazene-1,2-diyl)bis(4-cyanopentanoic acid)
DEAdcCM: Dicyanomethylene-coumarin
DEACM: 7-(diethylamino)-4-(hydroxymethyl)-coumarin
DOX: Doxorubicin Adriamycin
ER: Erythrocytes
FGF: Fibroblast growth factor
FITC-BSA: Fluorescein-labeled bovine serum albumin
5-FU: 5-Fluorouracil
FUS: Focused ultrasound
FA: Folic acid
Gd: Gadolinium
GSN: Gelsolin
GSH: Glutathione
GC: Glycol chitosan
AuNR: Gold nanorod
GNR: Gold nanorod
AuNS: Gold nanoshells
HA: Hyaluronic acid
Cy: Heptamethyl cyanine dye
HIFU: High-intensity focused ultrasound
HFUS: High-frequency ultrasound
HFLIU: High-frequency low-intensity ultrasound
InTBOD-Cl: Incorporating boron-dipyrromethene
ICG: Indocyanine green
INS: Insulin
IOL: Intraocular lens
LCST: Lower critical solution temperature
LFUS: Low-frequency ultrasound
LD: Levodopa
LIFU: Low-intensity focused ultrasound
LTSL: Lysolipid-based thermosensitive liposomes
MCH: Magnetic chitosan hydrogel
MLM: Magnetic lipid microcapsules
MNPs: Magnetic nanoparticles
MAN-TK-DOX: Mannose-thione-adriamycin complex
MSN: Mesoporous silica nanoparticle
nGO: Nanographene oxide
NIR: Near-infrared
NIPAM: N-isopropylacrylamide
NI: Nitroimidazole
MBAA: N-N-methylenebisacrylamide
LAM: N-lauroylalanine methyl ester
PTX: Paclitaxel
PFC: Perfluorocarbons
PFH: Perfluorohexane
PDE: Photodynamic effect
PDT: Photodynamic therapy
PTCE: Photothermal conversion efficiency
PTE: Photothermal effect
PTT: Photothermal therapy
P(AAm-co-AN)-g-PEG-LA: Polyacrylamide-co-acrylonitrile-polyethylene glycol-lipoic acid
PCL: Poly (ε-caprolactone)
PDA: Polydopamine
PEG: Polyethylene glycol
PEO-b-P(DEA-stat-MEMA): Poly(ethylene oxide)-block-poly(2-(diethylamino)ethyl methacrylate)-stat-poly(methyl methacrylate)
PDPA: Poly(diisopropylamino ethylene glycol)methacrylate
PMA: Poly(2-methacryloyloxyethylphosphorylcholine-azobenzylmethacrylamide)
PLA: Polylactic acid
PLGA: Poly Lactic-co-Glycolic Acid
PNIPAM: Poly(N-isopropylacrylamide)
P(NIPAM-co-VP): Poly(N-isopropylacrylamide-vinyl-2-pyrrolidone
PTHPMA: Poly(tetrahydropyranosyl methacrylate)
PVH: Poly(vinyl acetic acid-acrylic acid 2-hydroxyethyl ester)
PCO: Posterior capsule opacification
PpIX: Protoporphyrin IX
PPa: Pyropheophorbide a
ROS: Reactive oxygen species
Rh.B: Rhodamine B
RFP: Rifampicin
SDE: Sonodynamic effect
SPION: Superparamagnetic iron oxide nanoparticles
SDT: Sonodynamic therapy
TA: Tannic acid
TCPP: Tetrakis (4-carboxyphenyl)porphyrin
TTX: Tetrodotoxin
TL: Thioketal linkers
TK: Thioketal
TAEA: Tris(2-aminoethyl) amine
UCNPs: Upconversion nanoparticles
US: Ultrasound
UV: Ultraviolet
UCST: Upper critical solution temperature
Vis light: Visible light
VP: Verteporfin
XG: Xanthan gum
